# Rigorous characterization of urinary extracellular vesicles (uEVs) in the low centrifugation pellet - a neglected source for uEVs

**DOI:** 10.1038/s41598-020-60619-w

**Published:** 2020-02-28

**Authors:** Luca Musante, Sai Vineela Bontha, Sabrina La Salvia, Angela Fernandez-Piñeros, Joanne Lannigan, Thu H. Le, Valeria Mas, Uta Erdbrügger

**Affiliations:** 1 Division of Nephrology and Center for Immunity, Inflammation and Regenerative Medicine, Department of Medicine, University of Virginia, Charlottesville, VA USA; 2 Transplant Research Institute, James D. Eason Transplant Institute, School of Medicine, University of Tennessee Health Science Center, Memphis, TN USA; 3 School of Medicine, Flow Cytometry Core, University of Virginia, Charlottesville, VA USA; 4 Department of Medicine, Division of Nephrology, University of Rochester Medical Center, Rochester, NY USA

**Keywords:** Biomarkers, Diagnostic markers, Predictive markers, Prognostic markers

## Abstract

Urinary extracellular vesicles (uEVs) provide bio-markers for kidney and urogenital diseases. Centrifugation is the most common method used to enrich uEVs. However, a majority of studies to date have focused on the ultracentrifugation pellet, potentially losing a novel source of important biomarkers that could be obtained at lower centrifugation. Thus, the aim of this study is to rigorously characterize for the first time uEVs in the low speed pellet and determine the minimal volume of urine required for proteomic analysis (≥9.0 mL urine) and gene ontology classification identified 75% of the protein as extracellular exosomes. Cryo-Transmission Electron Microscopy (≥3.0 mL urine) provided evidence of a heterogeneous population of EVs for size and morphology independent of uromodulin filaments. Western blot detected several specific uEV kidney and EV markers (≥4.5 mL urine per lane). microRNAs quantification by qPCR was possible with urine volume as low as 0.5 mL. Particle enumeration with tunable resistive pulse sensing, nano particles tracking analysis and single EV high throughput imaging flow cytometry are possible starting from 0.5 and 3.0 mL of urine respectively. This work characterizes a neglected source of uEVs and provides guidance with regard to volume of urine necessary to carry out multi-omic studies and reveals novel aspects of uEV analysis such as autofluorescence of podocyte origin.

## Introduction

Urinary extracellular vesicles (uEVs) are a medley of exosomes, exosome-like vesicles and microparticles/microvesicles^1–4^. Confusing nomenclature aside^5,6^, all uEVs secreted in urine transport proteins, nucleic acid and small metabolites from all epithelial cells forming the nephron and lower urinary tract^7,8^. Thus, uEVs have become a valuable source of biomarkers for identifying any changes in the physio- pathological state of their parental cell. Moreover, uEVs are also bio-activators in renal diseases^9,10^. The most common method in use to enrich uEVs is a 2 or 3 step centrifugation protocol^11–13^. While it has been commonly discarded, the pellet obtained at relative low centrifugation force has proved to be an additional source of uEVs^14,15^. However this pellet has not been thoroughly characterized.

In addition, the concomitant presence of multiple biomarker in uEVs offers the possibility to integrate multi-omic data analysis to better understand mechanism and possibly identify key role molecules implicated in the onset and progression of the disease^16^. However, no study has reported the amount of volume of urine that is necessary to collect to support multiple analyses. Hence, this study aims to: (1) estimate the minimum volume of urine necessary to yield uEVs for characterization according to both minimal information for studies of extracellular vesicles (MISEV)^17^ and downstream analysis applying a very rigorous approach using several control sets for each analysis; (2) test the limit of detection of the techniques employed for downstream analysis and EV characterization before and after elimination of Tamm Horsfall protein (THP) - also known as uromodulin (UMOD) - the most abundant protein in the urine presenting one of the main technical challenges related to uEV isolation^18,19^; and (3) provide a rigorous analysis of uEVs in this low centrifugation pellet previously neglected, discuss specific uEV characteristics and interactions with Tamm Horsfall protein and a naturally occurring autofluorescence in urine.

## Results

Urinary extracellular vesiscles (uEVs) were enriched by centrifugation at relative centrifugation force (RCF) of 21,100 g (P21) from 0.5, 1.0, 1.5, 3.0, 4.5, 9.0 and 13.5 mL of urine respectively. An overview/summary of the study is schematized in Supplementary Fig. S1.

### Depletion of tamm horsfall protein (THP)

**Figure 1:**
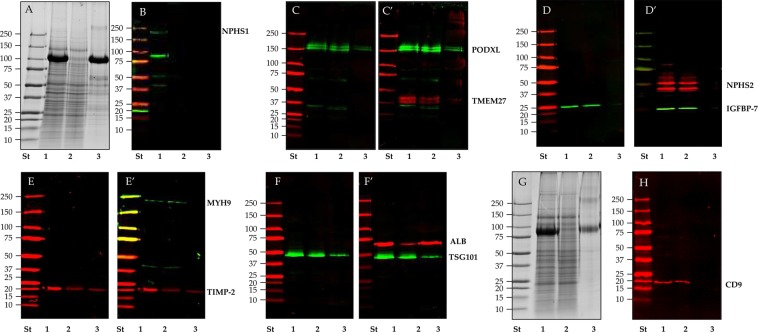
SDS-polyacrylamide gel electrophoresis (SDS-PAGE) and western blot (WB) analysis of P21 pellet after TCEP reduction in healthy donors. Pellets P21 (lane 1), P21^TCEP^ (lane 2) and SN21^TCEP^ (lane 3) originated from 9 (**A**,**C**–**H**) and 20 ml (**B**) of urine were loaded in each lane and stained with colloidal Coomassie (**A**,**G**). Nitrocellulose membranes were hybridized respectively with anti: podocalyxin (PODXL) (**C**) and collectrin (TMEM27) (**C’**); Insulin-like growth factor binding protein 7 (IGFBP-7) (**D**) and podocin (NPHS2) (**D’**); Tissue inhibitor of metalloproteinases 2 (TIMP-2) (**E**) and myosin 9 (MYH9) (**E’**); Tumor susceptibility gene 101 (TSG101) (**F**) and human serum albumin (ALB) (**F’**); Nephrin (NPHS1) (**B**) and CD9 antigen (CD9) (**H**). No reducing condition (-DTT) for CD9 WB (**H**) and respective protein patter gel (**G**). After the first acquisition the same membranes in panel C (PODXL), **D** (IGFBP-7), **E** (TIMP-2) and F (TSG101) were incubated again with anti TMEM 27(**C’**), anti NPHS2 (**D’**), anti MYH9 (**E’**) and ALB (**F’**).

THP was depleted by Tris (2-carboxyethyl) phosphine hydrochloride (TCEP-HCl) followed by a second centrifugation step at the same RCF and time (P21^TCEP^). The bulk of THP was released in the supernatant (SN21^TCEP^) (Fig. 1A,G) quickly and independently from the amount of THP (Supplementary Figs. S2 and S3). Western blot analysis confirmed that the majority of the signal for tested kidney markers like podocalyxin (PODXL, Fig. 1C), collectrin (TMEM27, Fig. 1C**’**), podocin (NPHS2, Fig. 1D**’**), Insulin-like growth factor binding protein 7 (IGFBP-7, Fig. 1D) and myosin-9 (MYH9 Fig. 1E**’)** while nephrin antigenicity (NPHS1 Fig. 1B) was lost after TCEP reduction. EVs marker like CD9 (Fig. 1H) were recovered mainly in the pellet P21^TCEP^ rather than in the SN21^TCEP^. Exceptions were tissue inhibitor of metalloproteases 2 (TIMP-2, Fig. 1E) and another EV marker: tumor susceptibility gene 101 protein (TSG101, Fig. 1F) which were detectable in smaller quantities in the SN21^TCEP^. Conversely, human serum albumin (ALB, Fig. 1F**’**) and THP (Fig. 1A) were mostly present in the SN21^TCEP^ .

### uEV morphology, counting and sizing

#### Cryogenic transmission electron microscopy (cryo-TEM)

**Figure 2:**
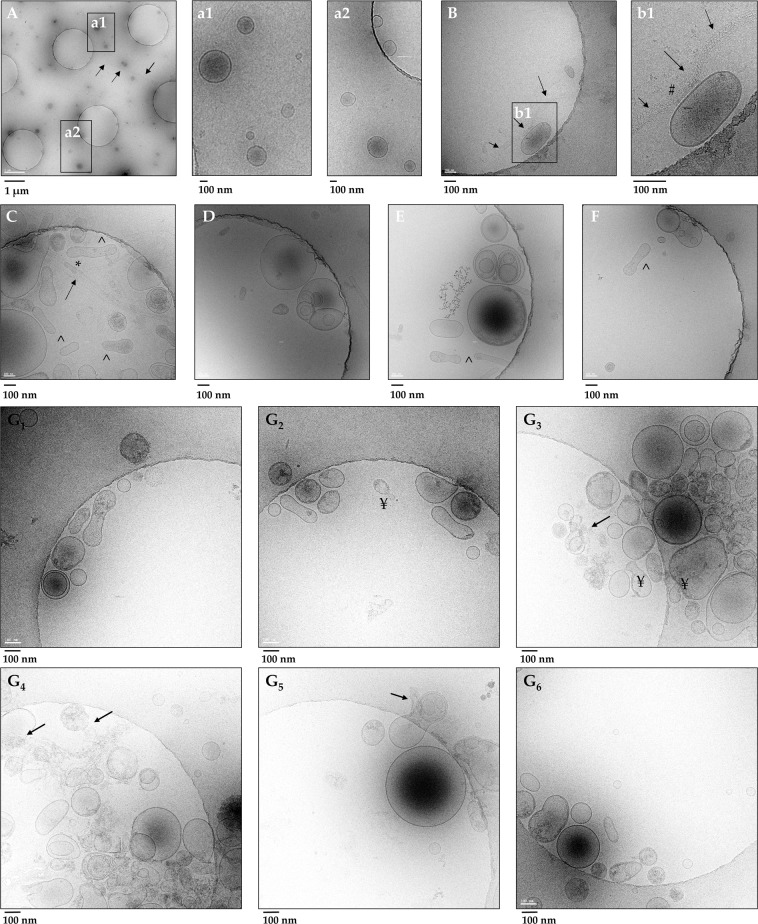
Gallery of Cryo-TEM images of urinary EVs recovered in the relative low centrifugation pellet P21 before and after TCEP reduction (P21^TCEP^). A heterogeneous population of uEVs was observed including single layered vesicles and multiyered structure with two or more inner small vesicles encapsulated inside bigger vesicles before (**A**–**F**) and after TCEP treatment (**G1**–**G6**). Tamm-Horsfall protein (THP) long polymeric filaments (indicated by arrows) (**A**) either engulfing (*) (**C**) or adsorbing (#) vesicles (**B** and **b1**) were also visible. No filaments of THP were visible (**G1**–**G6**) after TCEP reduction.

Pellet P21 from 3.0 mL of urine showed a heterogeneous population of uEVs for size and morphology (Fig. 2A). Enlarged images (Fig. 2a1,a2) showed vesicles of 60–250 nm diameter not associated with any THP filaments (arrows, Fig. 2A,B,b1,C) which can apparently entrap (*) **(**Fig. 2C) and/or bind vesicles (#) (Fig. 2b1). Vesicles are delimited by a clear double electrodense phospholipidic bilayer membrane mostly round in shape although some elongated/flattened vesicles (**^**, Fig. 2E,F) are also visible. Vesicles can be electro dense and present a more complex structure with either some granular matter or multi-layered structures with 1 or more smaller vesicles within it (Fig. 2D–F).

Cryo-TEM analysis performed on the pellet P21^TCEP^ THP depleted (Fig. 2G1–G6) confirmed the presence of a population of uEVs heterogeneous both for size (40–250 nm) and morphology (round, flattened electro dense, electro negative). In addition to multi composite EV structures, rupture of the plasma membrane, release of amorphous internal content (**arrow**), and bulging small vesicles from bigger ones (¥, Fig. 2G2,G3) can be seen, extending the repertoire of EV morphology.

#### Particle size distribution and number in tunable resistive pulse sensing (TRSP)

**Figure 3:**
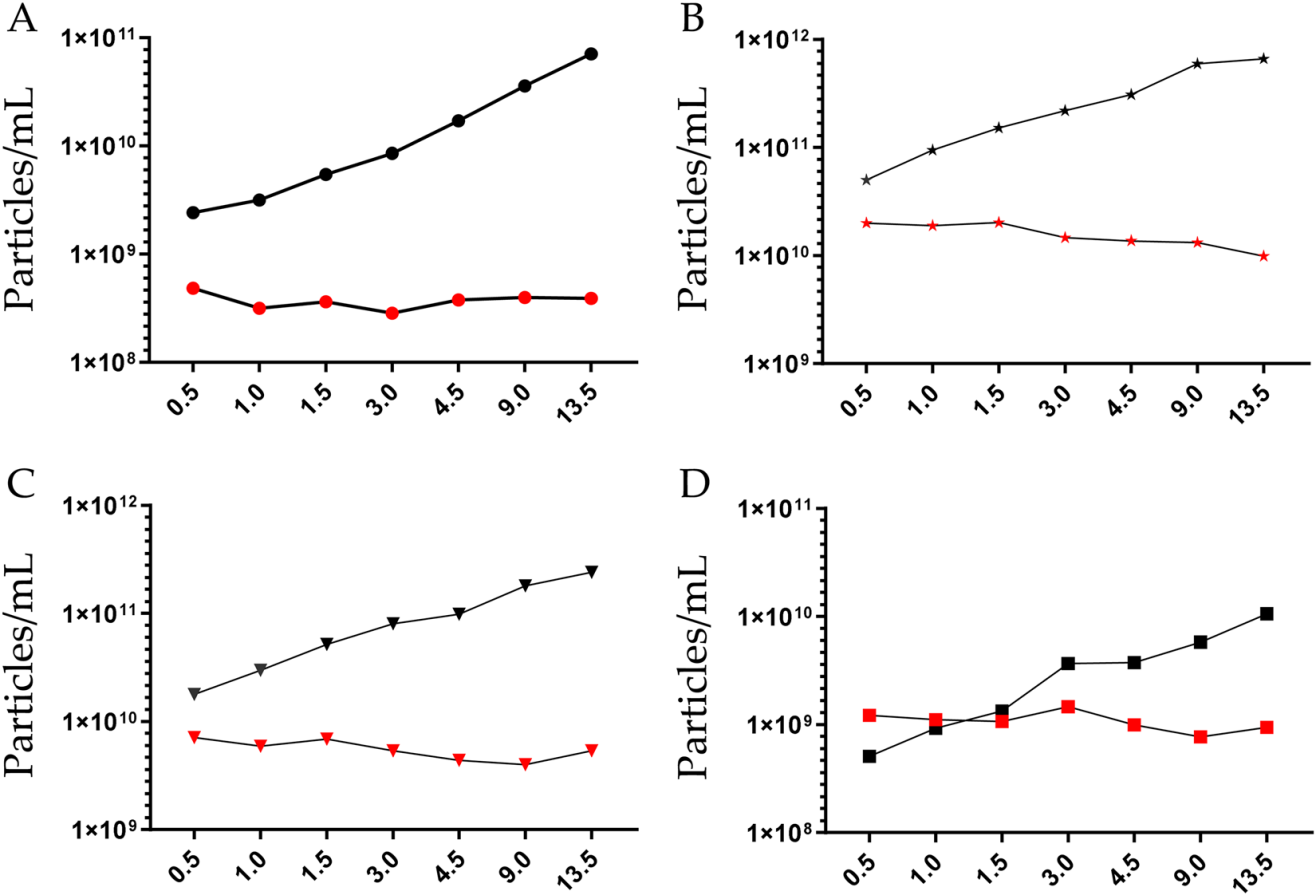
Particle size distribution and concentration. (**A**) Tunable resistive pulse sensing of P21 pellet; (**B**) Nanoparticle tracking analysis (NTA) of P21 pellet; **C** NTA of P21^TCEP^ pellet and (**D**) P21^TCEP^ supernatant. Black marks represent the particle concentration of re-solubilized pellet, red marks refer to the particles concentration per mL of urine. X- axis represent volume of urine (mL) processed to obtain the uEV pellet. Pellets for TRPS were re-solubilized in 100 μL of PBS-0.1μm (**A**). Pellets for NTA were resolubilized in 200 μL of 10 mM Tris-HCl pH8.8 + 4 mM TCEP- for P21 (**B**) and P21^TCEP^ (**C**) pellets and 1.2 mL 110 mM Tris-HCl pH8.8 + 4 mM TCEP for SN21^TCEP^ (**D**).

TRPS was employed to estimate both particle size distribution (PSD) and number. A nanopore membrane NP300 (analysis range 150–900 nm) was selected and two calibration particle standards SPK 200B (mean diameter 210 nm, mode diameter 200 nm) and CPC400E (mean diameter 340 nm, mode diameter 330 nm) respectively were used (Supplementary Table S1). Membrane stretch (46.84 mm), voltage (0.40 V), pressure (2,4 and 8 mbar) and sample dilution settings were experimentally established in order to have a linear particle rate (Supplementary Fig. S4), minimizing nanopore clogging whilst having a satisfactory blockade height of >0.05 nA (Supplementary Fig. S5). PSD was similarly independent of the volume of urine used to enrich uEVs (Supplementary Fig. S6). A moderate shift of 14 nm (mean) and 10 nm (mode) was seen when samples were calibrated with 200 nm (SPK200B) and 330 nm (CPC400E) standard particles respectively. As expected, the particle number increased with the volume of urine processed (Fig. 3A) for an estimated average urine concentration of 3.73 × 10^8^ ± 6.40 × 10^7^ (SPK200B calibration standard) and 2.04 × 10^8^ ± 3.55 × 10^7^ (CPC400B calibration standard) particles per mL of urine with a coefficient of variation of 17.1% and 17.4% respectively. Enumeration was possible with urine volume of 0.5 mL.

#### Nano tracking analysis (NTA) of particle size distribution before and after TCEP treatment

Size and particle concentration of P21, P21^TCEP^and SN21^TCEP^were measured by nanoparticle tracking analysis (NTA). As previously seen for TRPS, the PSD and particle concentration results were similar, independent of the volume of urine for P21, P21^TCEP^ and SN21^TCEP^ (Supplementary Fig. S7A–C). Not surprisingly, the particle number increased with the volume of urine processed (Fig. 3B, Supplementary Table S2) for an estimated average urinary concentration of 1.58 × 10^10^ ± 3.97 × 10^9^ particles per mL of urine with a coefficient of variation of 25.1%. After P21 TCEP reduction the particle number of P21^TCEP^ was 5.57 × 10^9^ ± 1.18 × 10^9^ particles per mL of urine with a coefficient of variation of 21.1% (Fig. 3C**;** Supplementary Table S3) and SN21^TCEP^ was 1.08 × 10^9^ ± 2.21 × 10^8^ particles per mL of urine with a coefficient of variation of 20.4% (Fig. 3D; Supplementary Table S4**)**. PSD was the same for P21, P21^TCEP^ and SN21^TCEP^ (Supplementary Fig. S7D). Taking into account that there was a minimal signal detection for uEV markers (Fig. 1), the relatively high SN21^TCEP^ particle concentration might come from THP. Enumeration was possible with urine volume of 0.5 mL.

### uEV cargo analysis

#### EV Proteomic analysis by mass spectrometry

A bottom-up proteomic approach was adopted to determine the protein composition of uEV pellet P21^TCEP^ without THP (Supplementary Fig. S8A). Overall, we found 1254 non-redundant gene name proteins with 2 or more unique peptides and 99% protein confidence (Supplementary Table S5**)**. We compared our data set with the list of the proteins deposited in the most recently updated (Version 4.1 8 15 2018) vesiclepedia repository and the subset of protein identifications specific for uEVs (Supplementary Table S5).

We found that 92.5% of our hits were common to the vesiclepedia data sets (Supplementary Fig. S8B). The gene ontology clustering applying the PANTHER algorithm showed that the protein distribution per cellular component and molecular function was substantially the same for P21^TCEP^ and vesiclepedia data sets (Supplementary Fig. S9,A–C). In the subcategories P21^TCEP^ showed less nucleus hits (GO:0005634) and more vacuole proteins (GO:0005773; Supplementary Fig. S9,A2–C2). The membrane sub-categories revealed a good representation of endosome membrane proteins (GO:0010008; 45.2% of the hits in uEV P21^TCEP^, 41.9% in uEV vesiclepedia and 30.1% in EV vesiclepedia; Supplementary Fig. S9,A3–C3) and apical plasma membrane proteins (GO: 0016324; 53.3% in uEV P21^TCEP^, 46.5% in uEV vesiclepedia and 25.3 in EVs vesiclepedia; Supplementary Fig. S9,A4–C4).

Analysis of the molecular function was in line with the cellular component (Supplementary Fig. S10,A–C,A1–C1). Some differences were found in some of the subcategories, such as: nucleic acid binding protein (GO:0003676; uEVP21^TCEP^ 48.3%; uEV vesiclepedia 77.2% and EV vesiclepedia 83.8%), nucleosome binding (GO:00031491; uEVsP21^TCEP^ 0.0%, uEV vesiclepedia 14.3% and EVs vesiclepedia13.9%) and actin filament binding (G0:0051015; uEVs P21^TCEP^ 69.2%; uEV vesiclepedia 35.8% and EVs vesiclepedia 13.9%; Supplementary Fig. S10, A2–C2,A3–C3).

Analysis of GO terms with DAVID recapitulate the results obtained with PANTHER. DAVID algorithm showed that the cellular component annotation with the highest percentage of proteins and low P value was extracellular exosome (GO:0070062; Supplementary Fig. S11A). Molecular function and KEGG pathway analysis (Supplementary Fig. S11B,C) highlighted the key role of these proteins in metabolic pathways, endocytosis and actin cytoskeleton regulation. Finally, in spite of the TCEP denaturation step to eliminate uromodulin, THP was still present in the MS list of the proteins identified. Interestingly, among the 15 unique tryptic peptides we found 2 peptides which matched the amino acid sequence of the domain between the catalytic cleavage - serine S in position 589 and the serine in position 614 bound to the glycophosphatidylinositol (GPI) anchor (Supplementary Fig. S12**)** [43].

#### Protein pattern and EV protein analysis by WB

**Figure 4:**
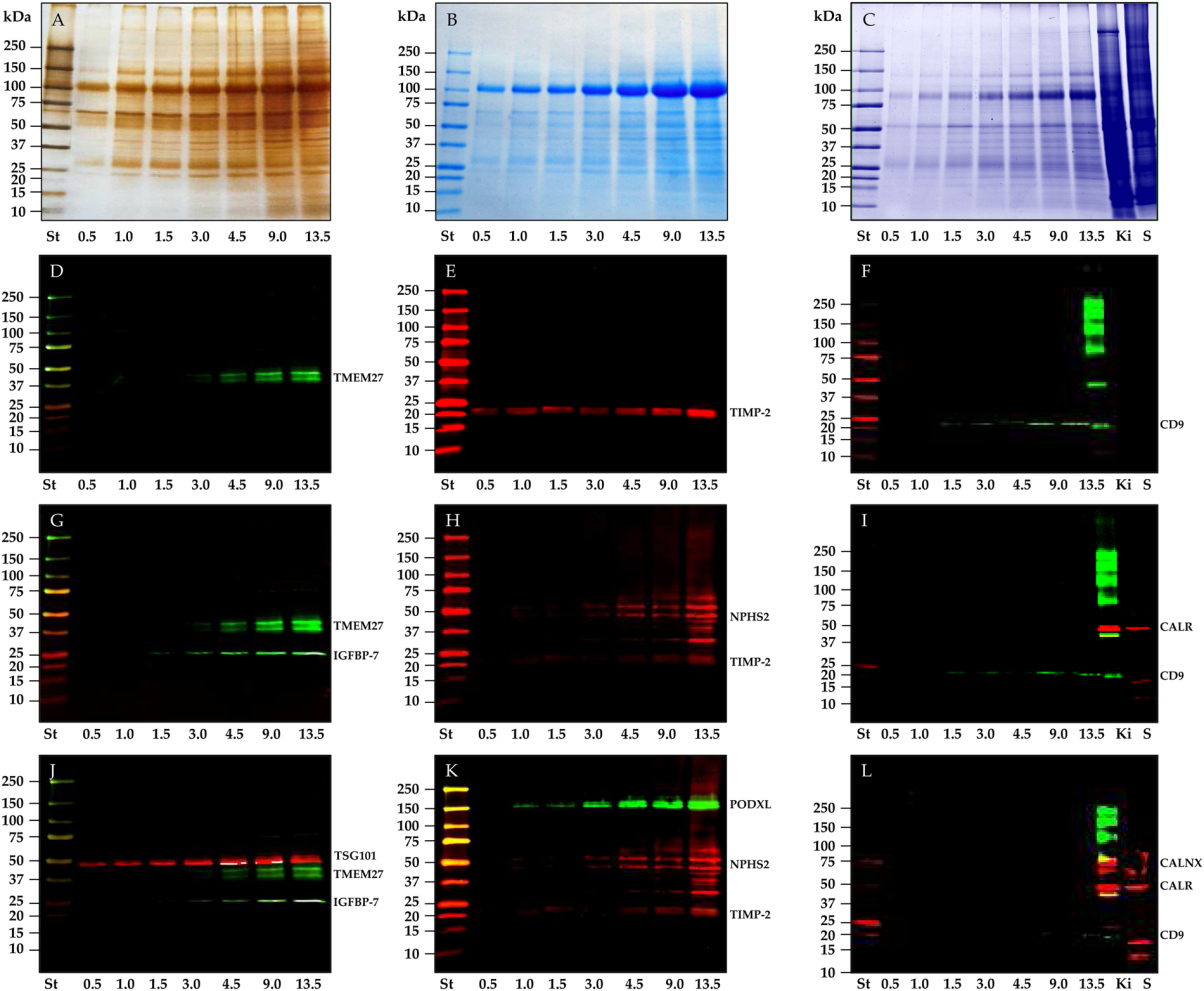
SDS-polyacrylamide gel electrophoresis (SDS-PAGE) and western blot (WB) analysis of P21 pellet. The whole pellet - originated from different volumes - was loaded in each lane and stained with either silver nitrate (**A**) or colloidal Coomassie (**B**,**C**). Nitrocellulose membranes were hybridized respectively with: collectrin (TMEM27) (**D**) and tissue inhibitor of metalloproteinases 2 (TIMP-2) and CD9. After the first image acquisition the same membranes were incubated again with insulin-like growth factor binding protein 7 (IGFBP-7) (**G**), podocin (NHPS2) (**H**) and calreticulin (CALR) (**I**) respectively. Finally membranes were incubated a 3^rd^ time with tumor susceptibility gene 101 (TSG101) (**J**), podocalyxin (PODXL) (**K**) and calnexin (**L**) respectively. Samples for gel in C and western blots in (**F**,**I**,**L**) were run without DTT. K rat kidney, S Saliva pellet 4.600 g.

Electrophoresis separation of P21 pellets obtained from 3 independent urine collections showed a very similar protein pattern for both silver staining (Fig. 4A) and Coomassie staining (Fig. 4B,C). THP was found to be the most abundant protein, the amount of which increased proportionally with increasing volume of urine.

Western blot analysis was performed to evaluate both EV markers according to the MISEV guidelines^18^ and markers of interest for downstream analysis. Tumor susceptibility gene 101 (TSG101, Fig. 4J), as part of the Endosomal Sorting Complex Required for Transport (ESCRT) machinery and CD9 (Fig. 4F), as it is one of the three tetraspanins, were selected as positive controls. Calreticulin (CALR, Fig. 4I) and calnexin (CALX, Fig. 4L) were targeted to exclude potential contaminants mimicking EVs from the intra cellular membrane compartments (endoplasmic reticulum). Two positive samples - rat kidney and saliva epithelial cells, were added to check the antibodies reactivity and cross-species specificity respectively. We did not detect either CALX or CALR at the level of sensitivity set for the acquisition; this suggests no major contaminations from cellular debris and intracellular membranes. Podocalyxin (PODXL, Fig. 4K), podocin (NHPS2, Fig. 4H) and collectrin (TMEM27, Fig. 4D) were selected as nephron-specific markers originating from podocyte, proximal and distal tubule cells respectively. Insulin-like growth factor binding protein 7 (IGFBP-7, Fig. 4G) and tissue inhibitor of metalloproteinases 2 (TIMP-2, Fig. 4E), which are secreted soluble proteins as part of the uEV proteome, are of great interest as biomarkers to predict the risk of developing acute kidney injury (AKI). All the antibodies have good specificity to recognize their own antigen at the right molecular weight with the exception of podocin, which shows a higher molecular weight band consistent with the ubiquitinated isoform^20^. The lower molecular weight fragment could be a splicing isoform lacking the PHB domain^21^. Most antibodies (TMEM-27, IGFB-7, CD9 and NPHS2) detected the respective antigen in P21 starting from 4.5 mL of urine, while PODXL, TSG101 and TIMP-2 could be found in as little as 1.0 and 0.5 mL of urine respectively.

#### EV Surface protein analysis by imaging flow cytometry (iFC)

iFC was employed as a tool for high-throughput single EV targeted protein analysis to analyse the surface distribution of uEV markers, namely, podocalyxin (PODXL), collectrin (TMEM27), insulin-like growth factor binding protein 7 (IGFBP-7), tissue inhibitor of metalloproteinases 2 (TIMP-2) and annexin V (AV). The antibody clones used in this analysis were also used to detect the same antigens in western blot. Analysis and gating strategy were established utilizing: buffer only, buffer plus reagents, buffer plus uEVs only and detergent lysis (Supplementary Figs. S13 and S14). Molecules of equivalent soluble fluorochrome (MESF) beads were used when available as a tool to provide standardized comparable results with different flow cytometry platforms (Supplementary Fig. S15). The analysis of the uEVs showed a unique natural auto-fluorescence proportional to the amount of uEVs (Supplementary Fig. S16) with a peak of emission in the red (CH 11) for camera 2, which captures the emission from both 405 nm and 640 nm excitation lasers, and for for camera 1(CH 5) which captures the emission for both the 488 nm and 561 nm excitation lasers. Since AV-APC emission is in CH 11, we created an additional gate to delimit this autofluorescence (AF). We also used the whole set of uEVs (0.5–13.5 mL) to evaluate if the median fluorescence intensity (MFI) was stable with the decrease of the particle counts (Supplementary Fig. S17), demonstrating that increased particle concentration did generate coincident (or aggregate) events. Application of morphology and intensity masks for the highest volume (13.5 mL) combined with the spot count feature on the positive gate for each antigen confirmed that the majority of the events are single events (Supplementary Fig. S17). Detergent lysis by Triton X-100 at concentration of 0.8% for 30 minutes at room temperature reduced (68.2%) the particle concentration in both TRPS (Supplementary Fig. S14A,B,C,E; Supplementary Table S7) and iFC. Concentration of PODXL, AV and AF (CH 11) decreased by 64.4%, 72.0% and 96.9% respectively (Supplementary Fig. S14F–H).

**Figure 5:**
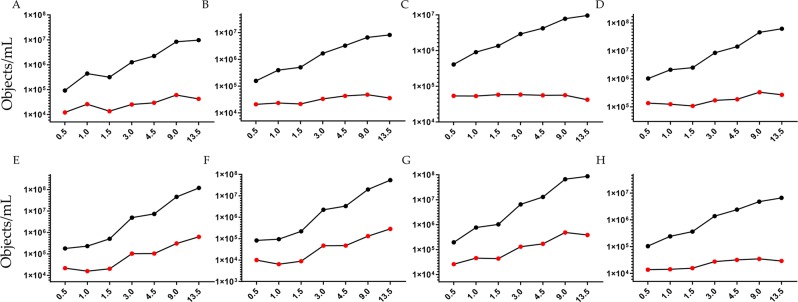
Imaging flow cytometry quantification. Concentration obtained by gating positive events (Supplementary Fig. S13) of: (**A**) IGFBP7; (**B**) TIMP2; (**C**) TMEM27; (**D**) AnnexinV; (**E**) Autofluorescence emission in channel 5; (**F**) Auto fluorescence emission in channe 11; (**G**) PODXL (**F**) PODXL without autofluorescence excluded applying the Boolean or logic function: “Not AF gate Ch5 And Not AF gate Ch11”. Black marks particles concentration of re-solubilized pellets, red marks particles concentration per mL of urine. X-axis shows the volume of urine (mL) processed to obtain the uEVs pellet.

**Figure 6:**
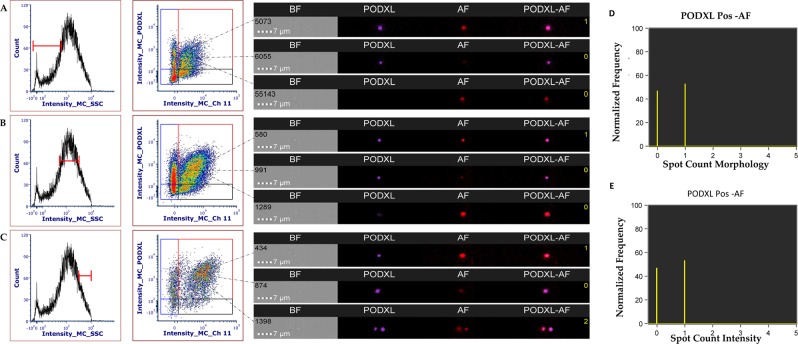
Imaging flow cytometry quantification single staining for PODXL. Co-detection of PODXL and AF emission in channel 11 at low (**A**), medium (**B**) and high (**C**) scatter based on the gate strategy in Supplementary Fig. S19. Application of morphology (**D**) and intensity (**E**) masks combined with the spot count feature show the number of coincidence event. Yellow digits in the image gallery indicate the number of events calculated by the spot feature.

Concentration of IGFBP7, TIMP2, TMEM27, PODXL, AV and AF (CH 5 and 11) increased proportionally to the volume of processed urine (Fig. 5, Supplementary Table S8). When results were reported as object per mL of urine we noticed that for IGFBP7, TIMP2, TMEM27 and AV positivity the coefficient of variation (CV) was 36.9%, 27.7%, 16.0% and 27.8% respectively (Fig. 5A–D). Conversely, for PODXL (Fig. 5G) the CV was 98.5%. This trend seems to follow the amount of AF particles detected in the uEVs only sample in channels 5 and 11 (Fig. 5E,F). When we applied the Boolean algorithm to exclude AF, the coefficient of variation dropped to 53.4% (Fig. 5H), thus suggesting a co-localization with PODXL. In fact among all possible combinations of antigens (Supplementary Fig. S18), the most prominent double staining occurred between PODXL and AF. Single staining for PODXL confirmed the co-localization of PODXL and AF at low (Fig. 6A), medium (Fig. 6B) and high (Fig. 6C) scatter intensities respectively. When we applied the Boolean mask for both for morphology and intensity, we found that more than 55% of the counts were double staining single events (Fig. 6D,E). Overall, as with the western blot, ideal urinary volume to process in order to enrich uEVs reaching a sufficient concentration to carry out a multiparametric characterization depends on the abundance or level of expression of the target marker. For all tested volumes we did not experience any swarming or coincidence effect. However, the presence of AF can be problematic when uEVs are enriched from large volume of urine (> 9.0 mL). Taking into account all these factors we conclude that for imaging flow cytometry, one of the more sensitive Flow Cytometric methods for EV detection, the best volume of urine for detection of uEV surface proteins is between 1.5 and 4.5 mL.

#### EV mi-RNA cargo analysis

**Figure 7:**
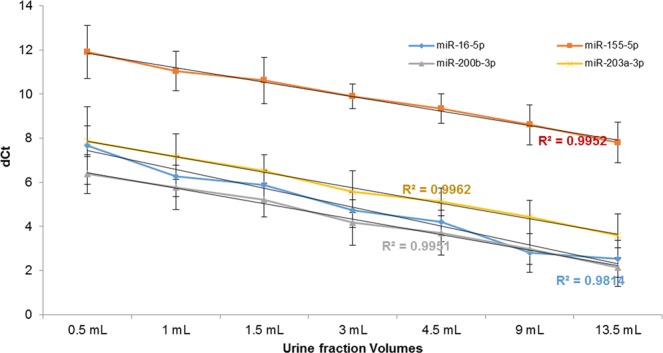
Real time quantitative PCR (qPCR) analysis of P21 pellet. RNA from the whole P21 pellet - originated from different volumes - was isolated and reverse-transcribed. qPCR analysis for miR-16 (blue), miR-155 (red), miR-200b (gray), and miR-203 (yellow) was performed and the relative cycle threshold values (dCt) were plotted for each of the volumes. The trend line with the R^2^ value indicates that the amount of miRNA increased with the increased volume of urine used for processing the pellet.

miRNA miR-16, miR-155, miR-200b, miR-203, have been previously reported to be found abundantly in cell-free fraction of urine from healthy volunteers by deep sequencing techniques [47–49]. These miRNAs were isolated from P21 pellet from urine collected on 3 different days from the same subject. Spike-in controls cel-miR-39 was added before the RNA extraction to normalize. The expression of miRNAs (dCT) was confirmed and it was observed to increase proportionally with increasing volume of urine that was processed to obtain the P21 urine supernatant pellet (Fig. 7). Even though the relative amount of miRNAs was lower in the 0.5 mL urine fraction, all the miRNAs tested were detectable in this lowest tested volume of urine fraction. It was also confirmed that these 4 miRNA that we tested were expressed in both P21 and the P21^TCEP^ and their expression level was higher than SN21 ^TCEP^ (Supplementary Fig. S20). These results are in line with WB and NTA analysis.

#### Characterization and recovery of uEV proteins from 8 healthy donors

**Figure 8:**
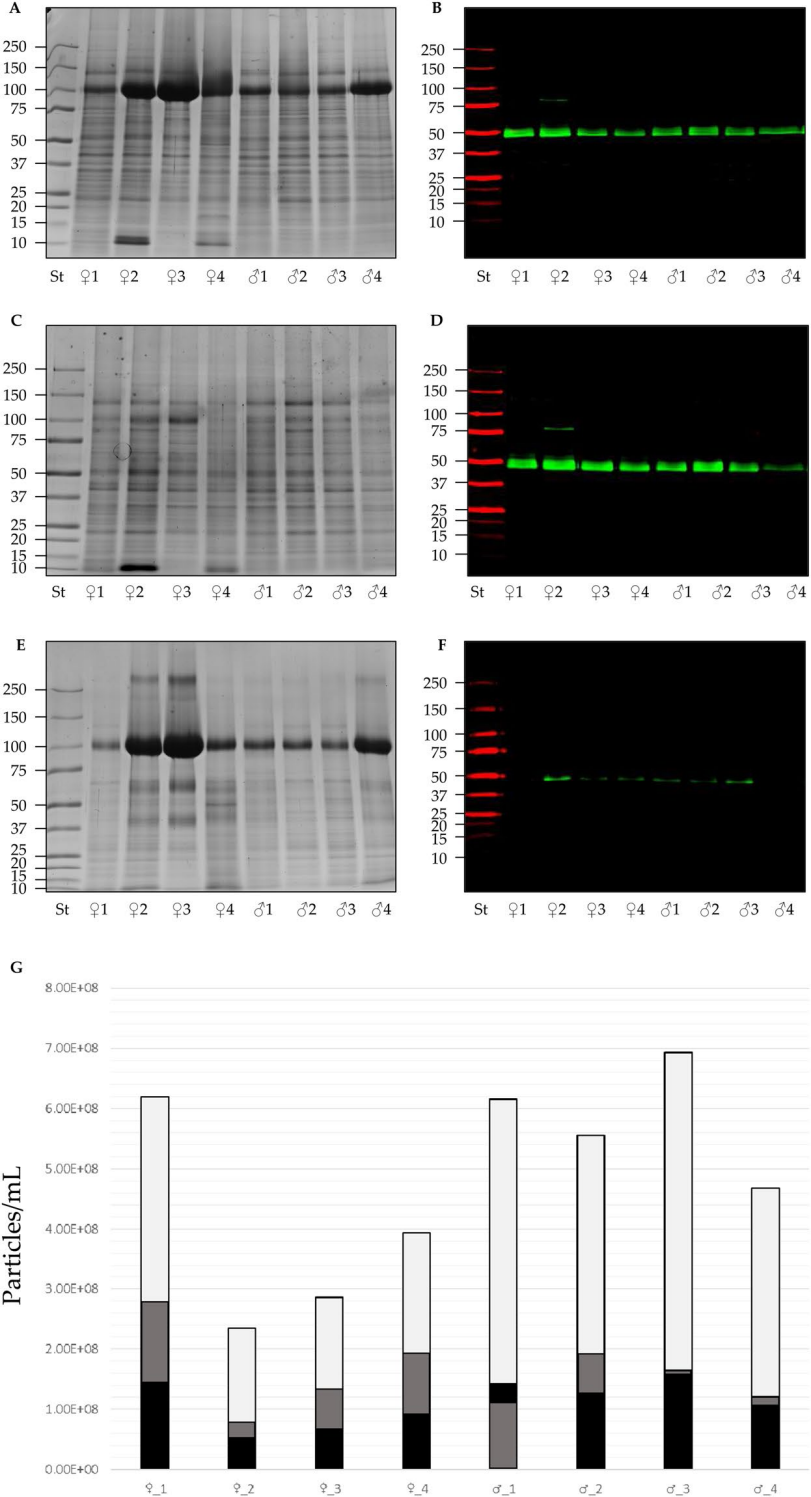
SDS-polyacrylamide gel electrophoresis (SDS-PAGE), western blot (WB) analysis and particles concentration of P21, P21^TCEP^ pellets and SN21^TCEP^ supernatants after TCEP reduction in healthy donors. Pellets and TCEP supernatant originated from 9 mL of urine from 4 female (lane1-4) and 4 male (lane 5–8) were loaded in each lane and stained with colloidal Coomassie or hybridized with anti tumor susceptibility gene 101 (TSG101) for P21 pellets (**A**,**B**), P21^TCEP^ pellets (**C**,**D**) and SN21^TCEP^ supernatants (**D**,**F**). Particle concentration measured with NTA (**G**) for P21 pellets (light gray), P21^TCEP^ pellets (grey) and SN21^TCEP^ supernatants (black).

In order to validate the minimal volume needed to characterize EVs, we studied 8 different healthy donors. We performed uEV analysis from 4 female and 4 male healthy donors and compared protein pattern and expression levels of TSG101. Based on the previous analysis for the proteomic validation we used a pellet (P21) from 9.0 mL of urine (Fig. 8A) treated with TCEP to eliminate THP interference (Fig. 8C,E). Western blot analysis of TSG101 confirmed the presence of markers in P21 (Fig. 8B**)** and in P21^TCEP^ (Fig. 8D) with minimal loss in SN21^TCEP^ (Fig. 8F). Particle concentration measured in NTA using a P21 originating from 3.0 mL of urine showed a moderate inter-individual variability in particle concentration and PDS. After TCEP reduction a higher variability for P21^TCEP^ and SN21^TCEP^ than P21 was noted (Fig. 8G, Supplementary Tables S9–S11).

## Discussion

Urinary extracellular vesicles have been extensively investigated for their novel role in cell- to cell communication, shuttling informative molecular cargo along the nephron and being a novel source of biomarkers^22–24^. We opted to analyse the first pellet of the differential centrifugation protocol as it has not been characterized rigorously and is mostly neglected by researchers. Our analysis also addresses the volume of urine necessary to provide EVs for multi-omic analysis on the same specimen, testing the limit of detection of instrument as well as the technical variability. In fact the 7 uEVs P21 pellets have to be considered as a septuple (7 replicas) of the same specimen. Our objective was to perform this rigorous characterization in keeping with “Minimal information for the study of EVs (MISEV) guidelines by the International Society for Extracellular Vesicles^17^. In particular, we applied several controls for each downstream analysis; this is especially important for flow cytometry analysis as many particles can mimic EVs. One of the major drawbacks of enriching uEVs is the co-sedimentation of THP, which can sediment readily at very low speed^25^ and entrap uEVs in its filaments^18^ or bind EVs^26^. Thus, one of our main goals was to reduce THP interference for MS analysis, RNA extraction and miRNA quantification, and NTA enumeration. We used Tris (2-carboxyethyl) phosphine hydrochloride (TCEP-HCl) as an alternative reducing agent to dithiothreitol (DTT) for its high reducing energy^27,28^. In fact, TCEP was able to quickly reduce the 24 disulfide bonds of THP at a 10 mM concentration. A key step for a successful release of THP in the supernatant (SN21^TCEP^) and recovery of uEVs in the centrifugation pellet (P21^TCEP^) is dilution prior to centrifugation. This step decreases the probability of unfolded THP to aggregate and precipitate following centrifugation^29^. Western blot analysis of EVs positive markers (TSG101, CD9) confirmed the expected recovery in P21^TCEP^ rather than the SN21^TCEP^ whilst two soluble protein like THP and ALB were released mainly in SN21^TCEP^. It is worth noting that two markers which have attracted a lot of interest in the prediction of acute kidney injury, IGFBP-7 and TIMP-2, seem to be differently affected by TCEP reduction. While IGFBP7 was completely retained in the pellet, TIMP2 was partially released in the supernatant after reduction. This suggests either a protective role of the EVs for some protein biomarkers (being packaged in EVs) or that the disulfide bonds do not play a key role in the tridimensional structure of the protein which is preserved. Unfortunately, no reports are available on the precise mechanism of their secretion as either soluble proteins (EV free) or adsorbed on the surface of the EV since both antigens were detected in flow cytometry. Further studies are required to establish the exact secretory pathway, the percentage of distribution between uEVs and the soluble urinary protein fraction. Of note, for many disease processes and biomarker discovery studies it is not known whether proteins like IGFBP-7 and TIMP-2 are superior biomarkers in soluble form or as packaged in EVs.

Our Cryo-TEM analysis is the first to investigate uEVs in cryo-TEM in the P21 pellet before and after denaturation of THP. We confirmed a wide variety (or heterogeneity) of different types of vesicles of different sizes (40–500 nm) and morphology (round, oval/flattened) with complex architecture made of a set of vesicles enclosed inside some larger ones independent of the presence of filament of uromodulin. This is consistent with Cryo-TEM analysis of EVs enriched from other biofluids, such as plasma^30,31^, synovial fluid^32^, ejaculate^33^ and urine^34^.

Particle size distribution and particle concentration determination was done with TRPS and NTA. Particles were detectable with as little as 0.5 mL urine for both TRPS and NTA with an overall coefficients of variation of 16% and 23% respectively. PSD distribution between TRPS and NTA was very close with an average diameter of 227.8 nm (TRPS) and 229.3 nm (NTA) and a mode diameter of 174.2 nm (TRPS) and 172.8 nm (NTA) respectively. However, NTA P21 detected a particle concentration two order of magnitude higher than TRPS. We note that it was beyond the aim of this study to compare NTA and TRPS. These two techniques are based on completely different principles and instrument settings. However, similar discrepancies were observed in other studies on different biofluids^32,33^.

We can explain this discrepancy in count numbers between TRPS and NTA on the basis that NTA is more sensitive to detect small particles made also of soluble proteins such as albumin, which can scatter light^34^. For example, the particle counts in the SN21^TCPE^, P21^TCEP^ and SN21^TCEP^ is still significant which might be due to the fact that THP can scatter and be detected as a particle like albumin.

Mass spectrometry protein analysis of P21^TCEP^ after THP elimination revealed a relatively complex protein composition with 1251 identified proteins. Gene ontology protein classification with both Panthers and DAVID algorithms^35–37^ showed that class distribution of proteins was not dissimilar from the vesiclepedia data sets. Some differences compared to the vesiclepedia data sets were noted when subcategorizing protein class with less nucleolar protein and more protein binding actin filaments. GO classification confirmed the presence of exosomes and apical plasma membrane vesicles carrying a variety of plasma membrane proteins specific to every type of epithelial cell forming the nephron. Podocalyxin (PODXL), nephrin (NPHS1), podocin (NPHS2) originating from podocytes and acquaporin-2 (AQP2) originating from collecting cells are just a few examples. Overall, independently from THP we confirmed that a low RCF can pellet different type of vesicles including exosomes (as defined by exosomes markers like TSG101 and CD9 for example) or small EVs^38–40^. Interestingly, our proteomic analysis revealed the presence of THP with a characteristic peptide pattern which include two peptides originating from the domain of the protein between the serine (S^614^) glycosylphosphatidylinositol (GPI) lipidation anchor site and hepsin cleavage site (^586^RFRS^589^)^41^. Hence, it is plausible that THP could be anchored to the membrane of uEVs secreted by the cells ascending Henle’s loop limb. Therefore, THP traces in uEV preparation might not be simply a mere contamination of the predominantly cleaved THP secreted form, but rather as part of the EV protein cargo.

Western blot analysis of P21 and P21^TCEP^ EV positive markers confirmed the presence of several EV markers such as TSG101 and tetraspanin CD9. The volume of urine to enrich uEVs and detect a marker of interest depends both on the abundance and the affinity of the antibody for the antigen. Ideally this should be shown by each study, however in many studies it has not been performed nor transparently reported. Overall, we estimate that 4.5 mL of urine is the minimum (or minimal) amount required to provide enough material to detect an antigen in western blot analysis but higher amount may be necessary for the detection of nephrin, where uEVs P21 were enriched from 20 mL of urine.

Imaging flow cytometry (iFC) was used as a high-throughput single EV analysis to detect uEV surface markers using multiple antigens. The advantages of iFC with respect to conventional flow cytometry have been already described^42,43^. iFC is one of the few highly sensitive flow-cytometers currently available for EV research. In particular iFC can provide increased sensitivity for the detection of smaller (including <100 nm) EVs which almost all conventional flow cytometers are incapable of detecting^44^. In addition, iFC provides robust population statistics and imaging confirmation of EVs utilizing a single technology^42,43^. We found an unexpected and never reported complication of the natural auto-fluorescence (AF) in the uEVs pellet without any reagents. Interestingly, AF was associated with EVs in general, but in particular with PODXL positive EVs. AF interferes with the counts of PODXL positive particles which resulted in a lack of proportionality to the amount of uEVs (Fig. 5). When the count was normalized by the volume of urine we found the coefficient of variation (CV 95%) was partially reduced when AF was excluded applying Boolean logic function (CV 54.3%) which offers a solution to correct for AF. However, AF was overall a rather complex factor which complicates the analysis and is amplified with the amount of volume of urine used to enrich EVs (Fig. 5E,F). For the aforementioned reason we think that a volume between 3 and 4.5 mL is optimal to perform a multiparametric analysis which includes 2 washing steps as per this study. This is the first report which highlights the difficulties associated with evaluations in the presence of AF in urinary EVs. This phenomenon is documented by some researchers^14^, but mostly ignored or not detected as it is below the instrument’s detection limit. However, the phenomenon of AF needs to be addressed as it can mimic artefactual EV counts by FC or lead to quenching of other fluorescent antibodies (or affect antibody performance). The biological relevance of the source of this AF is of interest and requires further studies.

EVs have been known as shuttles that also carry microRNAs that are crucial upstream regulators of gene expression. The role of urinary cell free miRNA in association with kidney disease and function has been reported^45–47^. It was observed that the detection was achieved with as little as 0.5 mL volume of urine for the lower abundant miRNA of the four miRNAs that were tested. This was indicated as an increased dCt in the reaction of RNA isolated from 0.5 mL of urine as compared to the other volumes. Furthermore, the same set of miRNAs was tested in the pellet P21 and P21^TCEP^. Again, the majority of the miRNA carried by uEVs was collected in the pellet after TCEP treatment with minimal release in the SN21^TCEP^, THP did not interfere with the RNA extraction and therefore TCEP treatment is not really necessary, as previously reported^48^. From our own experience it is likely that lower volumes could be sufficient for miRNA detection (data not published). However this particular study presented here did not assess volumes lower than 0.5 mL.

Although this study does not include any functional assay we believe that the utility of using a reducing agent to eliminate the interference of soluble proteins is useful for mass spectrometry analysis. More in general a reducing agent can have a detrimental impact on enzymatic activity^49^ particularly for those proteins which have key disulfide bonds in maintaining the tertiary and quaternary structure.

Finally, after uEV P21 characterization, we extended the analysis from uEVs enriched from different healthy donors. Sample protein patterns were very similar, even more so after THP removal. We investigated TSG101 as an EV marker because it was the most sensitive marker to detect the antigen in SN21^TCEP^, where only traces were detected. Particle concentration measured with NTA was consistent with a coefficient of variation (P21 CV 14.4%) in the same order of the technical variation.

In conclusion this study provides a detailed characterization of uEVs recovered at a centrifugation speed of 21,130 g with different urine volumes. We set up a new protocol to eliminate THP reducing the disulphide bonds with TCEP, which allowed recovery of the majority of uEVs in pellet P21^TCEP^. For each downstream analysis tool used, we had 7 replicas demonstrating the technical variability and repeatability of the enriched uEV samples from the different urine volumes studied. Proteomic analysis of P21^TCEP^ free of uromodulin confirmed the presence of a heterogeneous population of uEVs including smaller EVs such as exosomes (TSG101 and CD9 positive markers), supporting that the low centrifugation pellet is a rich source of EV biomarkers deriving from a heterogeneous group of EVs, not just larger EVs. We opted to analyse the first pellet of the differential centrifugation protocol as this approach has been ignored but can be equally informative without requiring the use of expensive instruments such as ultracentrifuges and associated rotors. We showed for the first time electron microscopy pictures of uEV P21 with and without THP filaments. We carried out multiparametric analysis with imaging flow cytometry, a very sensitive and high-throughput single EV analysis tool, describing the natural auto fluorescence of uEVs. This AF (not previously reported or overlooked) of the uEV prep influences the analysis of uEVs of podocyte origin, however we can offer a solution for an analysis algorithm to overcome this phenomenon of AF. We therefore conclude and summarize that the minimal volume of urine necessary to perform a multi –OMICs study and rigorous EVs characterization is: 9 mL for mass spectrometry; 1.0 mL for NTA TRPS; and 4.5 mL per lane Western blot but more urine volume could be necessary depending on antibody affinity and antigen abundancy on the uEV; 0.5 mL (and possibly lower) for qPCR, 3.0 mL for Cryo-TEM for P21and and 9.0 mL for P21^TCEP^, and between 1.5 and 4.5 mL for imaging flow cytometry.

This work makes significant contributions to the study of urine as a biofluid for EV research and provides uEV researchers guidance with regard to volume of urine necessary to carry out studies in keeping with MISEV guidelines and downstream analysis of interest. In addition, this work reveals novel aspects of uEV analysis such as AF in urine and interaction of uEV proteins with soluble proteins such as THP/Albumin, which need to be further studied and considered when doing uEV research.

## Materials and Methods

Additional detailed material and methods are provided in supplementary information. Chemical reagents were purchased from Sigma-Aldrich (Saint Louis, MO) unless otherwise specified.

### Urine samples

Urine samples were collected from a healthy volunteer aged 20–51 with no history of renal diseases, diabetes and hypertension. First morning void urine was processed within 3 h without adding any protease inhibitors. Written informed consent was obtained from the participant. This study was approved by The Research Ethics Committee of the University of Virginia (IRB HSR # 17192). All the experiments were performed in accordance with the declaration of Helsinki.

### Urinary extracellular vesicle enrichment

Urine was centrifuged at a Relative Centrifugal Force (RCF) of 4,600 g at max radius 168 mm (5000 rpm) in a TX-400 Sorvall ST16R (Thermo Fisher Scientific) swing bucket rotor (k Factor 9153) for 30 minutes at room temperature (RT) (braking set at 9). The supernatant 4,600 g (SN4,600) was centrifuged at max speed (15,000 rpm; RCF 21,130 g) for 30 minutes at room temperature in an Eppendorf microcentrifuge 5424 fix angle rotor (FA-45-24-11) (Eppendorf) using 1.5 mL microcentrifuge tubes (Axygen)) in volumes of 0.5, 1.0, 1.5, 3.0, 4.5, 9.0 and 13.5 mL urine. We refer to this pellet as P21. The supernatant was discarded and the tube refilled with 1.5 mL of SN4,600 for any volumes which were higher than 1.5 mL. Pellets were stored at −80 °C degrees as pellet and solubilized after thawing in 0.1 μm filtered (Minisart PES syringe filter code 16553-------K, Sartorious) phosphate buffered saline solution (PBS-**0.1μm**) pH 7.4 no Ca^2+^ and Mg^2+^ (Gibco, Life Technology) or electrophoresis solubilisation buffer (ESB).

### Depletion of tamm-horsfall protein from uEV P21

P21 pellets were solubilized in 100 μL of a solution made of 100 mM Tris-HCl (BioRad Laboratories) pH 8.8, 10 mM tris(2-carboxyethyl)phosphine hydrochloride (TCEP-HCl), 50 mM trehalose (Acros Organics), final pH 7.0 at RT from 15 minutes up to 1 hour depending on the experiment. Samples were vortexed 3 times within each time point. After the incubation samples were diluted up to 1.2 mL with 10 mM Tris-HCl pH8.8, 4 mM TCEP-HCl and centrifuged at 21,130 g (max speed 15,000 rpm). uEVs recovered in the pellet (pellet P21^TCEP^) were either stored at −80 °C degrees as pellet or solubilized in 10 mM Tris-HCl pH8.8, 4 mM TCEP-HCl (NTA measurements) or ESB. Supernatants (SN21^TCEP^) were transferred into a new tube and the protein content was precipitated by 20% (v/v) trichloraceticacid (TCA) and 0.08% (w/v) sodium deoxycholate (DOC)^50^.

### Protein assay, gel electrophoresis and western blot

Protein quantification was performed by Coomassie microassays^50,51^. SDS PAGE and western blot were performed as previously published^25^. Nitrocellulose membranes were saturated with Odyssey blocking buffer (Li-Cor Biosciences) and incubated in 0.5 μg/mL rabbit anti podocin (NPHS2) (Code P0372), 1.0 μg/mL rabbit anti myosin IIA (MYH9) (Code M8064) and 0.5 μg/mL rabbit anti tumor susceptibility gene 101 protein (TSG101) (Code T5701) (Sigma-Aldrich,); 1.0 μg/mL rabbit anti insulin-like growth factor binding protein 7 (IGFBP7) (Code ab171085) and 1.0 μg/mL rabbit anti nephrin (NPHS1) [Y17-R] (Code ab136894) (Abcam, Cambridge, UK); 1.0 μg/mL goat anti metalloproteinase inhibitor 2 (TIMP2) (codeAF971) and mouse anti human serum albumin (ALB) (code MAB1455) (R & D System, Minneapolis, MN); 1.0 μg/mL rabbit anti collectrin (TMEM) in house [27]; 1.0 μg/mL mouse anti CD9 antigen (CD9) (code HBM-CD9-100) (HansaBioMed Life Sciences); 1.0 μg/mL mouse anti podocalyxin (PODXL) (code NBP2-33108), 1.0 μg/mL rabbit anti calreticulin (CALR NB600-101) and 1.0 μg/mL rabbit anti calnexin (CANX) (code NB100-1965) (Novus Biologicals) overnight at room temperature (RT) in the Odyssey blocking buffer diluted 1:1 with in house PBS (10 mM sodium phosphate dibasic, 1.8 mM potassium phosphate monobasic, 137 mM sodium chloride, 2.7 mM potassium chloride) and 0.15% (v/v) Tween-20. After 3 × 10 minute washes in PBS-Tween (0.15%, v/v), membranes were incubated with goat anti mouse (code 925–68070 and/or 925–32210), goat anti rabbit (code 925–68071 and/or 925–32211) and donkey anti goat (code 925–68074) either red (displayed in red colour excitation 680 nm, emission 700 nm) or infrared (displayed in green colour excitation 780 nm, emission 800 nm) dye-coupled secondary antibody 0.1 μg/mL (Li-Cor Biosciences) in an Odyssey blocking solution diluted at 1:1 with PBS and 0.15% (v/v) Tween-20; 1 hour at RT. Acquisition of the fluorescent signal was performed by Odyssey infrared imaging system with resolution set at 169 µm (Li-Cor Biosciences). Image studio software version 2.1 (Li-Cor Biosciences) was used to analyse and export images.

### Mass spectrometry analysis (MS)

P21^TCEP^ was solubilized in 100 μL of 0.1 μm filtered PBS and delipidated by chloroform methanol^52^ before reduction, alkylation and trypsin digestion. The LC-MS/MS was performed on a Thermo Electron Velos Orbitrap ETD mass spectrometer (Thermo Fisher Scientific) in the biomolecular analysis facility at the University of Virginia (https://med.virginia.edu/biomolecular-analysis-facility/). The data were analysed by database searching using the Sequest search algorithm against Uniprot Human Proteome database. All search data were loaded into Scaffold (Version 4_3_4; www.proteomesoftware.com). To identify proteins previously found in EVs we outsourced the vesiclepedia repository^53^ (Version 4.1, 15 August 2018) (http://microvesicles.org/). Venn diagram was performed using the InteractiVenn tool (www.interactivenn.net)^54^. Protein identifications were categorized using two different open sources: Protein ANalysisTHrough Evolutionary Relationships (PANTHER version 14.0)^35^ and Database for Annotation, Visualization and Integrated Discovery (DAVID 6.8)^36,37^.

### Tunable resistive pulse sensing (TRPS)

TRSP measurements were performed with a gold qNano instrument (Izon Ltd) mounting a polyurethane nanopore membrane NP300 (analysis range 150–900 nm) (Izon Ltd). Electrolyte solution was made of PBS–0.1 μm supplemented with 0.03% (v/v) Tween-20 filtered with Minisart high flow hydrophilic 0.1 μm syringe filter (Sartorious). For the detergent lysis uEVs were incubated at room temperature for 30 minutes with 0.8% Triton X-100 and both uEVs and uEVs + triton were filtered with Minisart high flow hydrophilic 0.45 μm syringe filter (code 16533-------K, Sartorious). A NP200 (analysis range 85–500 nm) (Izon Ltd) nanopore membrane was used.

### Nanoparticle tracking analysis (NTA)

NTA was performed using the ZetaViewPMX 120 (Particle Metrix) configured with a 488 nm laser with a long wave-pass (LWP) cut-off filter (500 nm) and a sensitive CMOS camera 640 × 480 pixels. The instrument was set to a constant temperature of 25 °C, a sensitivity of 70, a shutter speed of 80 and a frame rate of 30 frames per second (fps). Each sample was measured at 11 different positions throughout the cell, with 5–7 cycles of readings at each position in order to have a minimum of 1000 traces. Automated report of the particles recording across the 11 positions were manually checked and any outlier position was removed to calculate particle concentration and distribution expressed by mode, median and mean.

### Cryo-transmission electron microscopy (Cryo-TEM)

Cryo-TEM was performed in the molecular electron microscopy core at the University of Virginia (https://med.virginia.edu/molecular-electron-microscopy-core/services/). Low speed centrifuged uEV P21 and P21^TCEP^ pellets were solubilized in 20 μL PBS–0.1 μm and applied to a glow-discharged, perforated carbon-coated grid (2/2-3C C-Flat; Protochips),. Low-dose images were collected at a nominal magnification of 29,000 × on the Tecnai F20 Twin transmission electron microscope operating at 120 kV. Digital micrographs were recorded on a Gatan US4000 charge-coupled device camera^55^.

### Imaging flow cytometry

Imaging flow cytometry was performed according to the methods described previously^42^ using a dual camera ImageStream Mark II operated by INSPIRE software (Luminex Corporation). The data acquisition was performed in the flow cytometry facility at the University of Virginia (https://med.virginia.edu/flow-cytometry-facility/). Fluorescence intensity calibration was performed using Quantum Cellular Molecules of Soluble Fluorochrome (MESF) kit beads (Bangs Laboratory, Inc. Fishers, IN). Data were analysed using IDEAS application software (version 6.02; Amnis/Luminex Corporation) and De Novo Software FCS Express Flow Cytometry Data Analysis (version 6.06.0022; (http://www.denovosoftware.com/). All the raw files were exported as.fcs files and are available at (FlowRepository.org; ID: FR-FCM-Z2AB)^56^.

### RNA extraction and analysis

RNA was isolated from P21 pellets of 0.5, 1.0, 1.5, 3.0, 4.5, 9.0, and 13.5 mL urine fractions that were resuspended in 0.1 mL of 1X PBS using miRNA serum/plasma kit (Qiagen). Cel-miR-39 was spiked in the qiazol solution as per manufacturer’s instructions. For the SN21^TCEP^ as well, the RNA was isolated using the same kit. RNA concentration was measured using Nanodrop 2000 (Thermo Fisher Scientific). Reverse transcription was performed using Taqman miRNA reverse transcriptase kit (Thermo Fisher Scientific). Primers for miR-16, miR-155, miR-200b, miR-203, and Cel-miR-39 were used for reverse transcription followed by real time QPCR (Quantstudio3, Thermo Fisher Scientific). The results were then exported and the Ct values for each of the miRNA were normalized against the spike-in control (Cel-miR-39) and the obtained dCt values for the urine P21 pellets from various fractions were compared.

### EV-TRACK

We have submitted all relevant data of our experiments to the EV-TRACK knowledgebase (EV-TRACK ID: EV190076)^57^.

## Supplementary information

Supplemental information Figures.

Supplemental information S1Table.

Supplemental information S2Table.

Supplemental information S3Table.

Supplemental information S4Table.

Supplemental information S5Table.

Supplemental information S6Table.

Supplemental information S7Table.

Supplemental information S8Table.

Supplemental information S9Table.

Supplemental information S10Table.

Supplemental information S11Table.
