# Supplementary material for: Rigorous characterization of urinary extracellular vesicles (uEVs) in the low centrifugation pellet - a neglected source for uEVs
Source: Sci Rep. 2020 Feb 28;10:3701. doi: 10.1038/s41598-020-60619-w (PMC7048852; doi:10.1038/s41598-020-60619-w)
Supplement: Supplementary file 1 — Supplemental information Figures. [file 41598_2020_60619_MOESM1_ESM.pdf]

Rigorous characterization of urinary extracellular vesicles (uEVs) in the low centrifugation pellet - a neglected source for uEVs

Luca Musante, Sai Vineela Bontha, Sabrina La Salvia, Angela Fernandez Piñeros, Joanne Lannigan, Thu H Le, Valeria Mas, Uta Erdbrügger

Supplementary Materials and Methods.

Chemical reagents were purchased from Sigma-Aldrich (Saint Louis, MO) unless otherwise specified.

Whole Saliva collection.

Whole saliva was collected from a healthy volunteer by spitting into sterile 50 mL tube and kept in ice until a final volume of 10 mL was obtained. Saliva was split between 2 tubes (5mL each) and centrifuged at 4,600g at max radius 168 mm (5000 rpm) in a TX-400 Sorvall ST16R (Thermo Fisher Scientific, Waltham, MA) swing bucket rotor (k Factor 9153) for 30 minutes at room temperature (RT) (braking set at 9).. Pellets were resuspended in PBS-0.1μm or 100 μL electrophoresis solubilization buffer (ESB) respectively.

Rat kidney microsome enrichment.

The experimental protocols were conducted in accordance with guidelines reviewed and approved by the University of Virginia Animal Care and Use Committee. Winstar Kyoto (WKY) rat (Harlan Tekland, Envigo) were housed in vivarium under controlled conditions: 12-h cycle of light and dark with; temperature (21.1 ± 1.0 °C) and humidity (60% ± 10%). Animals were allowed free access to food and water. Kidneys were obtained from 12 week old WKY rats. Animals were anesthetized with Nembutal (0.3 mL intraperitoneal), kidneys were carefully excised, followed by euthanization. Kidneys were minced with a razor blade and homogenized in 10 mL 10 mM Hepes pH 7.4, 0.2M mannitol , 50 mM sucrose, 1 mm EDTA 1 X SIGMAFAS

proteases inhibitor cocktail (Sigma-Aldrich code S8830) with a homogenizer set at 20,000 rpm (Tissue Tearor; Biospec)[1]. All operations were performed in a bath of ice. The homogenate was centrifuged at 800g (rpm) for 10 min at 4°C in in a TX-400 Sorvall ST16R (Thermo Fisher Scientific) swing bucket rotor (k Factor 9153) (braking set at 9).. The supernatant was centrifuged at 21,130g (15,000 rpm) for 30 min at 4°C in an Eppendorf microcentrifuge 5424 (Eppendorf) fix angle rotor (FA-45-24-11) using 1.5 mL microcentrifuge tubes (Axygen, Corning Inc,). Finally, a standard crude microsomal fraction was obtained by centrifugation using Beckman Coulter XL-80 ultracentrifuge and a type 90 Ti Rotor (Beckman Coulter) (k factor at maximum speed 25) at 116,000g (37,000rpm) for 90 min at 4°C in polycarbonate tubes (Beckman Coulter part number 355630) (max braking).. Microsomal pellet was resuspended in PBS-0.1µm or electrophoresis solubilization buffer (ESB)

Depletion of Tamm-Horsfall Protein from uEV P21

P21 pellets were solubilized in 100 µL of a solution made of 100 mM Tris-HCl (BioRad Laboratories) pH 8.8, 10 mM tris(2-carboxyethyl)phosphine hydrochloride (TCEP-HCl), 50 mM trehalose (Acros Organics), final pH 7.0 at RT from 15 minutes up to 1 hour depending on the experiment. Samples were vortexed 3 times within each time point. After the incubation samples were diluted up to 1.2 mL with 10 mM Tris-HCl pH 8.8, 4 mM TCEP-HCl and centrifuged at 21,130g (max speed 15,000rpm). uEVs recovered in the pellet (pellet $P21^{TCEP}$) were either stored at -80°C degrees as pellet or solubilized in 10 mM Tris-HCl pH8.8, 4 mM TCEP-HCl (NTA measurements) or ESB. Supernatants ($SN21^{TCEP}$) were transferred into a new tube and the protein content was precipitated by 20 % (v/v) trichloraceticacid (TCA) and 0.08 % (w/v) sodium deoxycholate (DOC)[2]. Briefly, 300 µL of 100 % (v/v) TCA + 0.4 % (w/v) DOC was added to 1.2 mL of $SN21^{TCEP}$, vortexed and incubated in ice for 30 minutes. Samples were then

centrifuged at max speed (15,000 rpm; RCF 21,130 g) for 30 minutes at 1°C. Supernatant was discarded and the pellet was added 800 μL 100 % (v/v) acetone and incubated at -20°C overnight and then centrifuged max speed (15,000 rpm; RCF 21,130 g) for 30 minutes at 1°C. Supernatants were discarded and the pellets dried in the fume hood for 10 minute to be finally solubilized in either ESB or of PBS-0.1μm respectively.

Protein assay, Gel electrophoresis and Western blot

Protein quantification was performed by Coomassie microassays[3]. The series of P21 pellets were solubilised in 40 μL of ESB made of: 6 M urea (BioRad Laboratories), 2 M thiourea, 5% (w/v) sodium dodecyl sulphate (SDS) (BioRad Laboratories), 40 mM Tris-HCl, pH 6.8, 0.5 mm ethylenediaminetetraacetic acid (EDTA) (BioRad Laboratories), 20% (v/v) glycerol and 50 mM dithiothreitol (DTT) (BioRad Laboratories). Samples were denaturized overnight at room temperature (RT). Proteins were separated by hand cast SDS-PAGE gradient gels (Resolving gel T= 5-20 % (w/v); C=2.6 %; Stacking gel T= 3.5 % (w/v); C=2.6 %) in 25 mM Tris (Bio-Rad Laboratories), 192 mM glycine (BioRad Laboratories) and 0.1 % (w/v) SDS (Bio-Rad Laboratories) buffer[4] and either stained with colloidal Coomassie G-250[5] or silver nitrate[6] or transferred onto a 0.45μm nitrocellulose membrane (Amersham Protean 0.45μm NC, Life Sciences) in a wet transfer system buffer made of 25 mM Tris, 192 mM glycine and 20 % (v/v) methanol for 2 hours at 200 mA per gel in ice bath[7].

Mass Spectrometry Analysis (MS)

$P21^{TCEP}$ was solubilized in 100 μL of 0.1 μm filtered PBS and delipidated by chloroform methanol[8]. Briefly, 400 μL of 100 % (v/v) methanol was added to the sample, vortexed and

centrifuged for 10 seconds at 9000g. Two hundred µL of chloroform were added, vigorously vortexed and centrifuged for 10 seconds at 9000g. Three hundred µL of deionized water was added, mixed vigorously and centrifuged for 5 minutes at 9000g. The aqueous upper phase was discarded and the interface protein layer was precipitated by adding 300 µL of 100 % (v/v) methanol and centrifuged for 10 minutes at max speed. The sample was reduced with 10 mM DTT in 0.1 M ammonium bicarbonate at room temperature for 0.5 h. The sample was then alkylated with 50 mM iodoacetamide in 0.1 M ammonium bicarbonate at room temperature for 0.5 h. Excess of iodoacetamide was quenched by adding 50 mM N-acetyl cysteine. The sample was then digested overnight at 37°C with 0.5 µg of trypsin MS grade (Promega) in 50 mM ammonium bicarbonate. Peptide cleaning was performed by mixing 20 µL Sera-Mag Hydrophylic and Sera-Mag Hydrophobic Magnetic Particles (GE Life Sciences 24152105050250 and 44152105050250). Briefly, the mixture was washed in 1 mL of HPLC-grade water four times using a magnetic rack (DynaMag, Thermo Fisher Scientific). After the last wash magnetic beads were resolubilized in 20 µL of 95 % (v/v) acetonitrile (ACN). Six µL of particle suspension was added to 5 µL of 2 µg/µL peptide sample in 95 % (v/v) ACN and mixed thoroughly The mixture was then allowed to settle for 8 min before being placed on the magnetic rack for an additional 2 min. The supernatant was carefully removed and 200 µL of 100% HPLC-grade ACN was added to wash the beads. The tube was returned to the magnetic rack and the mixture was allowed to settle for 2 min, and the supernatant wasted. Finally, peptides were eluted in 25 µL of 2% LC-MS ACN in LC-MS water.

The LC-MS/MS was performed on a Thermo Electron Velos Orbitrap ETD mass spectrometer (Thermo Fisher Scientific) system with an easy spray ion source connected to a Thermo 3 µm C18 Easy Spray 50 cm column (through pre-column) in the biomolecular analysis facility at the

University of Virginia (https://med.virginia.edu/biomolecular-analysis-facility/). The equivalent of 1.5 µg of protein digested by trypsin was injected and peptides eluted from the column by an acetonitrile/0.1 M acetic acid gradient at a flow rate of 0.3 µL/min over 3.0 hours. The nano spray ion source was operated at 1.9 kV. The digestion was analyzed using the rapid switching capability of the instrument acquiring a full scan mass spectrum to determine peptide molecular weights followed by 20 product ion spectra (MS/MS-Ion trap) to determine amino acid sequence in sequential scans over the gradient elution.

The data were analysed by database searching using the Sequest search algorithm against Uniprot Human Proteome database. All search data were loaded into Scaffold V 4_3_4 (www.proteomesoftware.com) with the following filters: 2 or greater unique peptides, parent mass 10 ppm, fragment tolerance mass 1.0 Da, variable modifications oxidized methionine (+16), carbamidomethyl cysteine (+57), propionamide (+71), 1 trypsin miss cleavage site and masses between 600 and 4000 Da. Scoring cut-offs were used to minimize false positives. Peptide Prophet score >90%, Protein Prophet score > 99%, Xcorr> 1.8 (+1), 2.2 (+2), 2.5 (+3), and 3.5 (+4 or greater).

Tunable Resistive Pulse Sensing (TRPS)

TRSP measurements were performed with a gold qNano instrument (Izon Ltd, Christchurch, New Zealand) mounting a polyurethane nanopore membrane NP300 (analysis range 150-900nm) (Izon Ltd). Multi pressure at 2,4 and 8 mBar respectively; membrane stretch 46.84 mm and voltage 0.30 V were applied to determine the particle concentration and size distribution. Detergent lysis measurements were performed with a NP200nanopore membrane (analysis range 85-500nm) (Izon Ltd). Multi pressure at 4,6 and 8 mBar membrane stretch 45.0 mm and voltage 0.52 V were respectively applied. Electrolyte solution was made of PBS-0.1µm supplemented

with 0.03 % (v/v) Tween-20 filtered with Minisart high flow hydrophilic 0.1 µm syringe filter (Sartorious). Current pulse signals were collected using Izon Control Suite 3.2 software (Izon Ltd). Low speed centrifuged uEVs were solubilized in the filtered electrolyte solution and diluted proportionally to the starting volume in order to perform the measurement with a similar particle rate. Polystyrene particle standards (SPK200B and CPC400B;IzonLtd.) were employed for calibration. Both uEV pellets and standards were measured with a minimum of 700 blockades. For the detergent lysis uEVs were incubated at room temperature for 30 minutes with 0.8 % Triton X-100 and both uEVs and uEVs + triton were filtered with Minisart high flow hydrophilic 0.45 µm syringe filter (code 16533-------K, Sartorious)

Nanoparticle Tracking Analysis (NTA)

NTA was performed using the ZetaViewPMX 120 (Particle Metrix) configured with a 488 nm laser with a long wave-pass (LWP) cut-off filter (500nm) and a sensitive CMOS camera 640 x 480 pixels. Each P21sample was diluted in 2 mL of 0.1 µm filtered (Minisart high flow hydrophilic 0.1 µm syringe filter Sartorious) deionized water (DI 18 MΩ/cm) to obtain a particle concentration between 1 x 10^7 and 1 x 10^8 particles/mL[9]. The instrument was set to a constant temperature of 25°C, a sensitivity of 70, a shutter speed of 80 and a frame rate of 30 frames per second (fps). Each sample was measured at 11 different positions throughout the cell, with 5-7 cycles of readings at each position in order to have a minimum of 1000 traces. Post-acquisition parameters were set to a minimum brightness of 30, a maximum size of 200 pixels, and a minimum size of 5 pixels. Automated cell quality control was checked using high quality deionized water (DI). Camera alignment and focus optimization was performed using polystyrene 100 nm beads (Applied Microspheres). Data analysis was performed with ZetaView 8.02.28 software provided by the manufacturer. Automated report of the particles recording

across the 11 positions were manually checked and any outlier position was removed to calculate particle concentration and distribution expressed by mode, median and mean.

Imaging Flow Cytometry.

Labelling of uEV surface protein was performed in conjunction with annexin V surface labelling. We used anti rabbit anti insulin-like growth factor binding protein 7 (IGFBP-7) (Code ab171085) (Abcam), conjugated with Rapid Alexa Fluor 488 (AF488; Lightning-Link Antibody, Protein & Peptide Labeling Kits code 332-0030, Expedeon); goat anti metalloproteinase inhibitor 2 (TIMP-2) (codeAF971) (R & D System,) conjugated with PE/ R-phycoerythrin conjugation kit (code ab102918 size 60μg) (Abcam), rabbit anti collectrin (TMEM-27) conjugated with Alexa Fluor 568 (AF568; Lightning-Link Antibody, Protein & Peptide Labeling Kits code 334-0030) and annexin V (code 640920) (Biolegend). Labelling of the antibodies: anti IGFBP-7 (0.603 μg/μL); anti TIMP-2 (1.0 μg/μL) and anti TMEM (1.14 μg/μL) with the respective kit was performed according to instructions. Briefly: 1 μL of LL-rapid modifier reagent was added for each 10 μL of antibody and added to the fluorescent dye vial. Conjugation was performed overnight at room temperature in the dark. After incubation 1 μL of LL-rapid quencher for 10 μL of antibody labelled was added.

uEV (P21) pellets were resolubilized in 50 μL PBS-0.1μm of antibody mix made of 1 μL IGFBP7-AF488, 1 μL TIMP2-PE, 1 μL TMEM27-AF568 and 1 μL of anti PODXL conjugated with Alexa Fluor 405 (code NBP2-33108AF405, Novus Biologicals, Littleton, CO) pre-centrifuged at max speed (15,000 rpm; RCF 21,130 g) for 30 minutes at RT in an Eppendorf microcentrifuge 5424 (Eppendorf) fix angle rotor in a 1.5 mL microcentrifuge tube (Axygen). Samples were incubated for 1 hour at RT in the dark. Incubation was stopped by adding 1 mL of PBS-0.1μm and centrifuged as above. uEV pellet was resuspended in 48 μL of 0.1 μm filtered

annexin V binding buffer (Catalogue number 556454; Becton Dickinson Biosciences) plus 2 µL of annexin V conjugated with allophycocyanin (APC) (code 640920, Biolegend) pre-centrifuged at max speed (15,000 rpm; RCF 21,130 g) for 30 minutes at RT in a 1.5 mLmicrocentrifuge tube (Axygen). Samples were incubated for 1 hour at RT in the dark. Incubation was stopped by adding 1 mL of filtered annexin V binding buffer and centrifuged as above. Finally, pellets were resolubilized in 50 µL of filtered annexin V binding buffer.

Imaging flow cytometry was performed according to the methods described previously[10] using a dual camera ImageStream Mark II operated by INSPIRE software (Luminex Corporation, Seattle, WA). The data acquisition was performed in the flow cytometry facility at the University of Virginia (https://med.virginia.edu/flow-cytometry-facility/). Briefly, fluorescent signals were excited using excitation lasers powers of 405 nm diode laser at 120mW; 488 nm solid state laser at 100 mW; 561 nm solid state laser at 200 mW; 642 nm diode laser at 150mW; side scatter (SSC) 785 nm diode laser set at 70 mW respectively. Bright field (BF) images were collected in CH 1 (camera 1) and CH9 (camera 2); fluorescent signals for AF488 was collected in CH 2 (camera 1, 480-560 nm filter), PE was detected in CH 3 (camera 1, 560-495 nm filter), AF568 was detected in CH4 (camera 1, 595-660nm), AF405 was detected in CH 7 (camera 2, 420-505 nm filter), APC was detected in CH 11 (camera 2, 630-720nm filter). All images were collected using the 60X magnification option. A total of 1000 events were collected for each single stained compensation control at the same settings, with the BF and SSC illumination turned off, in order to develop a compensation matrix to remove dye spectral overlap from each of the channels. All fully stained samples and controls were recorded for 3 minutes to insure equal volumes were collected for assessing contribution of background from the buffer Fluorescence intensity calibration was performed using Quantum Cellular Molecules of Soluble Fluorochrome (MESF)

kit beads (Bangs Laboratory, Inc.). Four fluorescent microsphere populations labelled with varying known amounts of R-PE, AF-488, APC for each channel respectively were acquired with the same laser powers used for the stained samples, with the exception of the SSC laser, which was reduced to 2 mW due to the size and material of the beads. After this calibration, MESF units were consequently assigned to each sample, allowing direct comparisons of data from run to run. Ultimately, all samples and calibration beads were acquired using the 60X magnification and the highest resolution setting (Low). Data were analysed using IDEAS Application v6.02 software (Amnis, Luminex Corporation) and De Novo Software FCS Express Flow Cytometry Data Analysis (version 6.06.0022; http://www.denovosoftware.com/). In each experiment, a compensation matrix was created using single color controls and was applied to all files. In the post-acquisition analysis two different "masks" were created by the IDEAS analysis software using a Boolean OR logic function of all 12 channel masks (M01-M12) according to the default masking algorithm. In brief, the first mask was created to circumvent the unexpected interference of uEV natural auto-fluorescence (AF). We set a new population using the Boolean logic function: "Not AF gate Ch5 And Not AF gate Ch11" to exclude the interference of AF from the analysis of PODXL. The second mask was generated to discriminate true single events from coincident particles by combining the spot count feature with a morphology and intensity mask of the channels of interest using the Boolean logic function: "Morphology (M07, PODXL) And Morphology (M11, AF)" and "Intensity (M07, PODXL 15-150) And Intensity (M11, AF 15-150). Image-based gating was evaluated with buffer only control, buffer plus reagents control (no uEVs), single staining acquisition, buffer plus uEVs only (no reagents) and detergent lysis. Lysis was carried out incubating uEVs with 0.8 % (w/v) Triton X-100 for 10 minutes at RT after

an initial 30 seconds vortex. All the raw files were exported as .fcs files and are available at (FlowRepository.org; ID: FR-FCM-Z2AB).

References

1. Graham, J.M. Preparation of crude subcellular fractions by differential centrifugation. *Scientific World Journal.*; **2**, 1638-4162 (2002).

2. Koontz, L. TCA precipitation. *Methods Enzymol*. **541**, 3-10 (2014).

3. Bradford, M.M. A rapid and sensitive method for the quantitation of microgram quantities of protein utilizing the principle of protein-dye binding. *Anal. Biochem*. **72**, 248–254 (1976).

4. Laemmli, U.K. Cleavage of structural proteins during the assembly of the head of bacteriophage T4. *Nature*. **227**, 680-685 (1970).

5. Candiano, G. *et al.* Blue Silver: A very Sensitive Colloidal Coomassie G-250 Staining for Proteome Analysis, *Electrophoresis*. **25**, 1327-1333 (2004).

6. Shevchenko, A. *et al*. Mass Spectrometric Sequencing of Proteins from Silver-Stained Polyacrylamide Gels. *Anal. Chem*. **68**, 850-858 (1996)..

7. Towbin, H., Staehelin, T. & Gordon, J. Electrophoretic Transfer of Proteins from Polyacrylamide Gels to Nitrocellulose Sheets - Procedure and some Applications. *Proc. Natl. Acad. Sci. USA*. **76**, 4350-4354 (1979).

8. Wessel, D. & Flügge, U.I. A method for the quantitative recovery of protein in dilute solution in the presence of detergents and lipids. *Anal. Biochem*. **138**, 141-143 (1984).

9. Mehdiani, A. *et al*. An Innovative Method for Exosome Quantification and Size Measurement *J Vis Exp.*;**17**,50974 (2015).

10. Lannigan, J. & Erdbruegger, U. Imaging flow cytometry for the characterization of extracellular vesicles. *Methods*. **112**, 55-67 (2017).

Supplementary Figure Legends

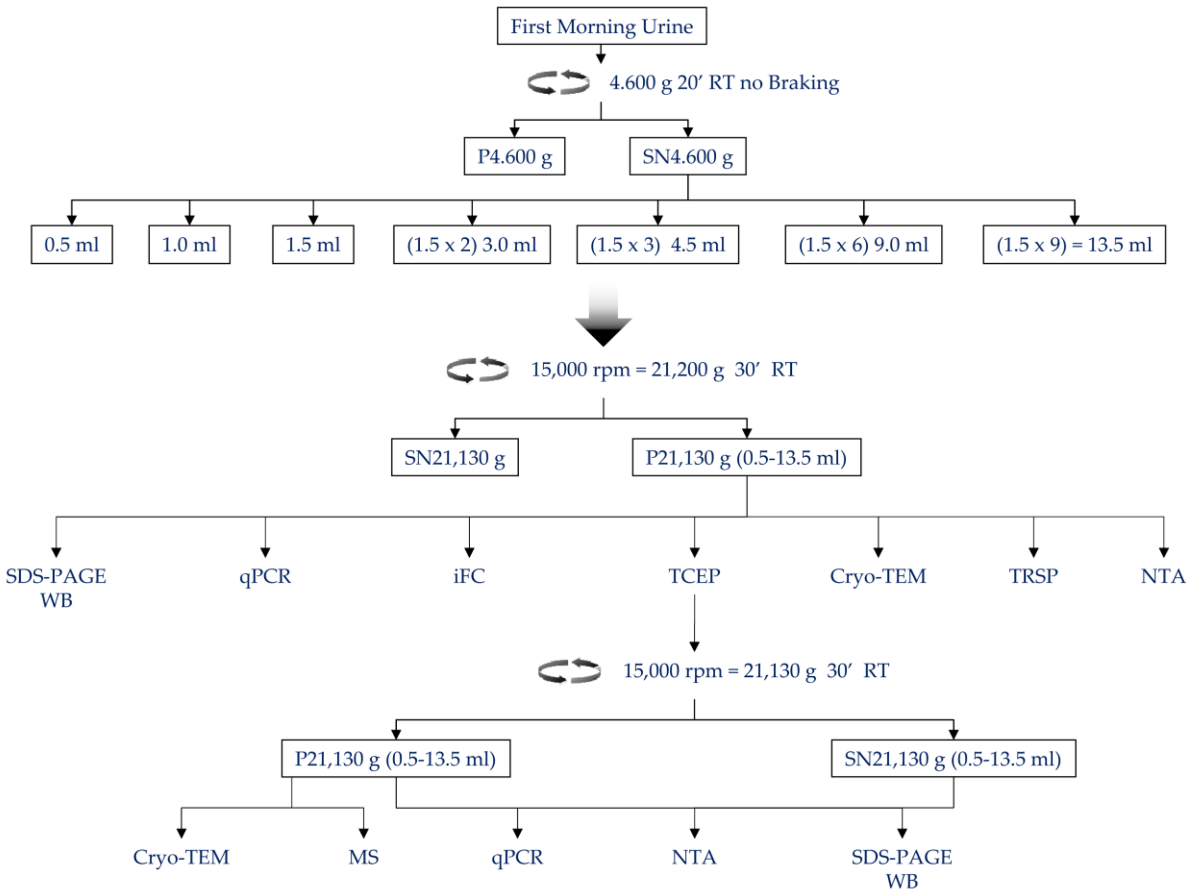

Supplementary Figure S1: **Urinary vesicle enrichment and analysis.** The workflow to isolate and fractionate urinary vesicles included two steps centrifugation at 4,600g and 21,200g (P21) respectively. For mass spectrometry analysis the bulky interference of THP in P21 was eliminated by disulphide bonds reduction with tris (2-carboxyethyl)phosphine hydrochloride TCEP followed by a third step centrifugation to recover uEVs.

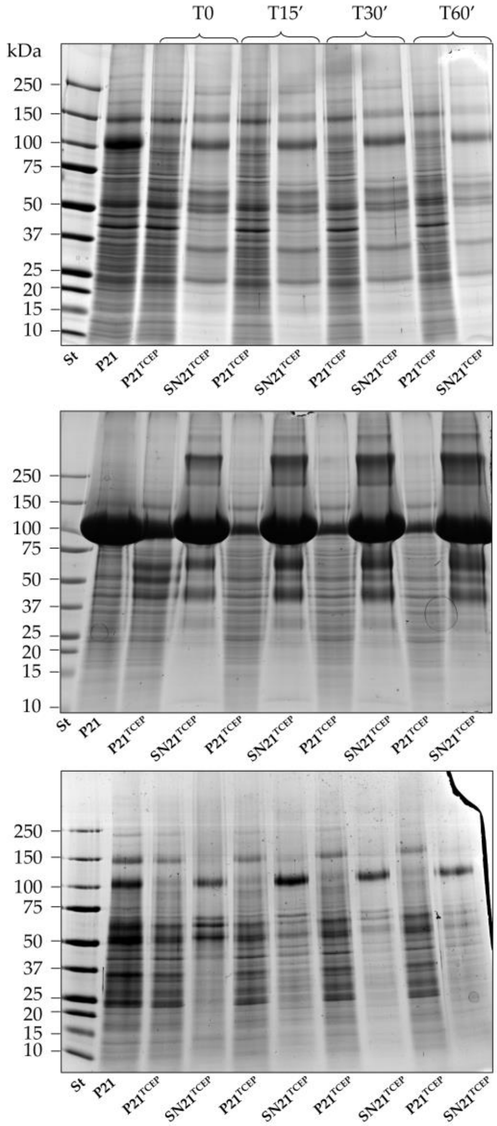

Supplementary Figure S2 Time course of TCEP reduction. Pellets P21 originated from 3 different collections was incubated for 60, 30, 15 minutes respectively. At the end of each time point samples were diluted adding 1.1 mL of 4mM TCEP in 10 mM Tris-HCL pH 8.8 and centrifuged at 21,000g for 30 minute at room temperature. For time 0 10 µL of 100 mM TCEP solution was added to the sample, diluted to 1.2 ml and centrifuged as described above.

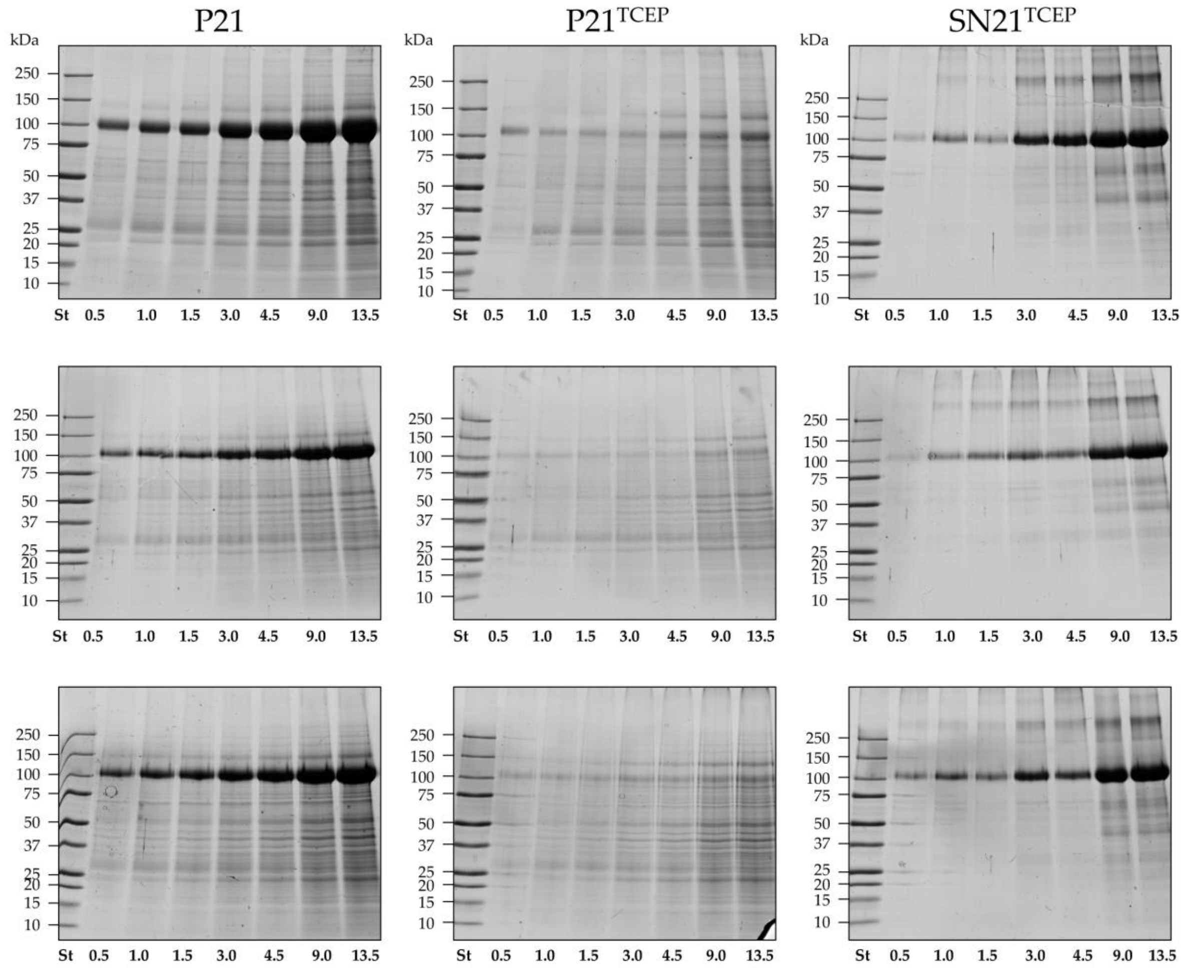

Supplementary Figure S3 THP dependence of the effect of the same concentration of TCEP and time. Pellets P21 originated from 0.5, 1.0. 1.5, 3.0, 4.5, 9.0 and 13.5 mL of urine from 3 different collections was incubated for 15 minutes with 10 mM TCEP. At the end of the incubation time samples were diluted adding 1.1 mL of 4mM TCEP in 10 mM Tris-HCL pH 8.8 and centrifuged at 21,000g for 30 minute at room temperature.

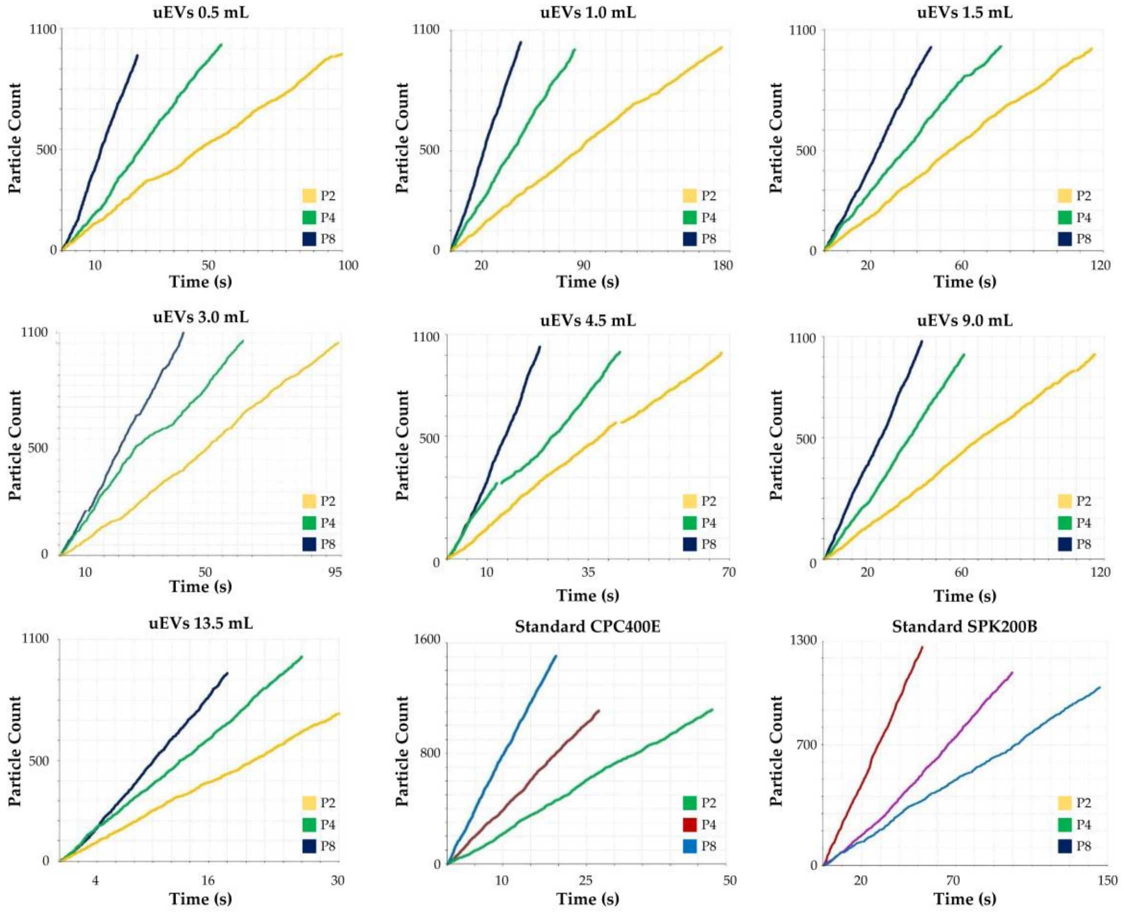

Supplementary Figure S4: **uEVs and calibration standards particle rate.** Each sample volume and particle standard was measured applying a pressure of 2,4 and 8 mBar. Most of the measurements were linear with a moderate drift of the particle rate for P4 in sample P21_3.0 mL (D) and P4 in sample P21_3.0 mL (D) after restoring nanopore from blocking events.

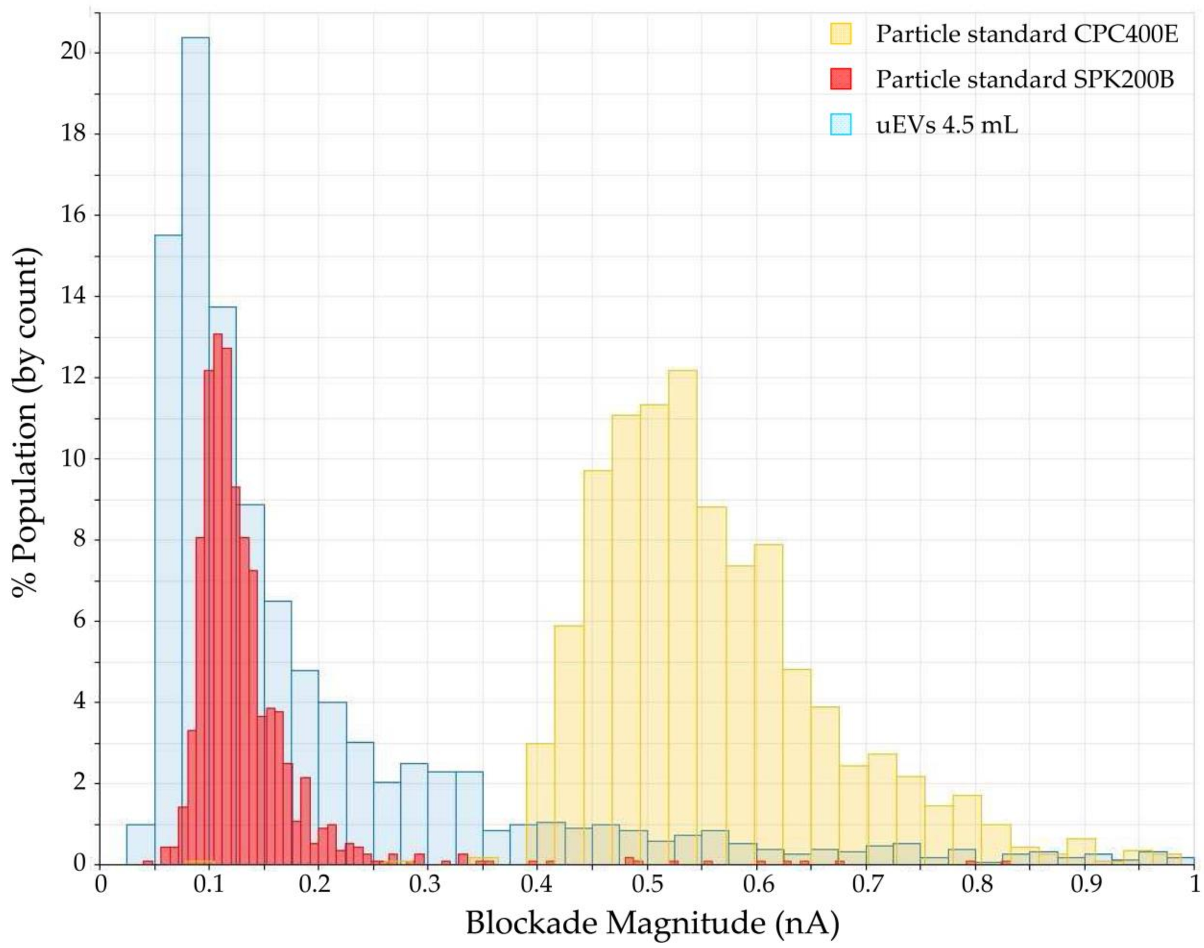

Supplementary Figure S5: **uEV and calibration standards blockade rate.** Blockade frequency example for uEVs P21 from 4.5 mL and calibration standards 200 nm (SPK200B) and 400 nm (CPC400E) measured at pressure of 4 mbar. Calibration standards show a Gaussian distribution of the blockades while the asymmetry of the uEVs sample reflects the broad polydispersity which extended below the cut off limit of 0.05 nA

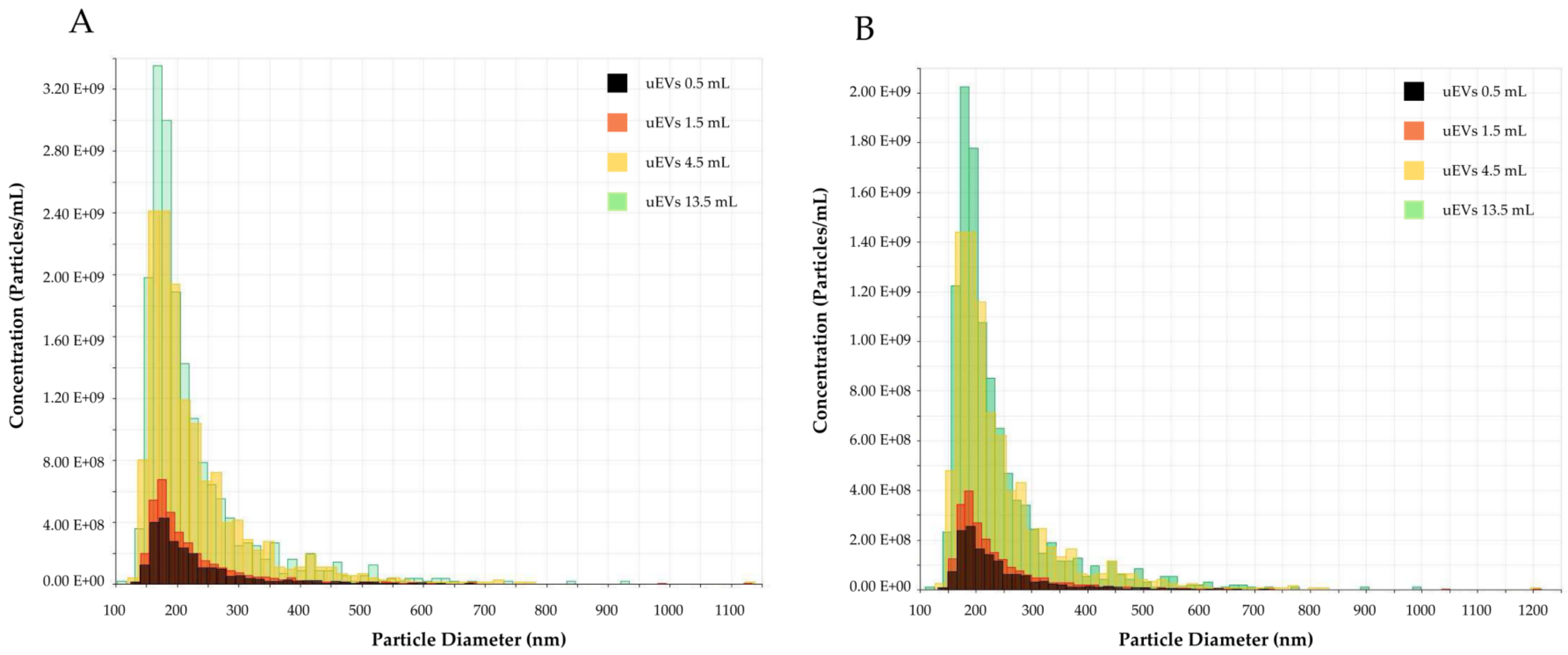

Supplementary Figure S6: **Particle size distribution (PSD) of P21 uEV yielded from 0.5, 1.5, 4.4 and 13.5 mL of urine.** PSD obtained using 200 nm (SPK200B) (Panel A) and 330 nm (CPC400E) (Panel B) calibration standard particle

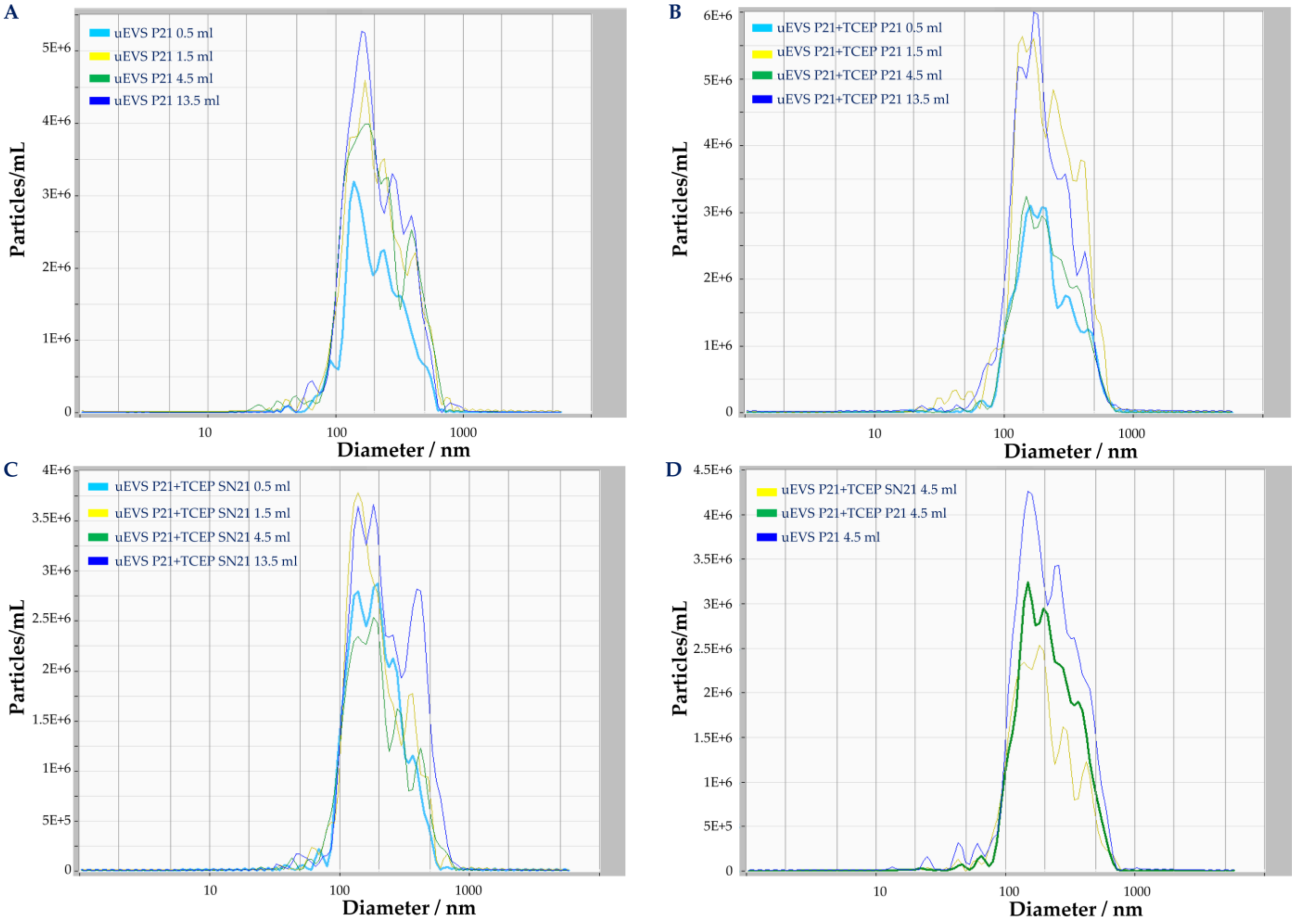

Supplementary Figure S7 Particle size distribution (PSD) measured in NTA of P21 uEVs and $P21^{TCEP}$ pellets and $SN21^{TCEP}$ supernatant. PSD yielded from 0.5, 1.5, 4.4 and 13.5 mL of urine were superimpose to evaluate potential change in the size distribution for P21 (panel A); $P21^{TCEP}$ (panel B); $SN21^{TCEP}$ (panel C). The 3 fraction for the pellet P21 originate from 4.5 mL was overlapped to check changing in the PSD after TCEP treatment (Panel D).

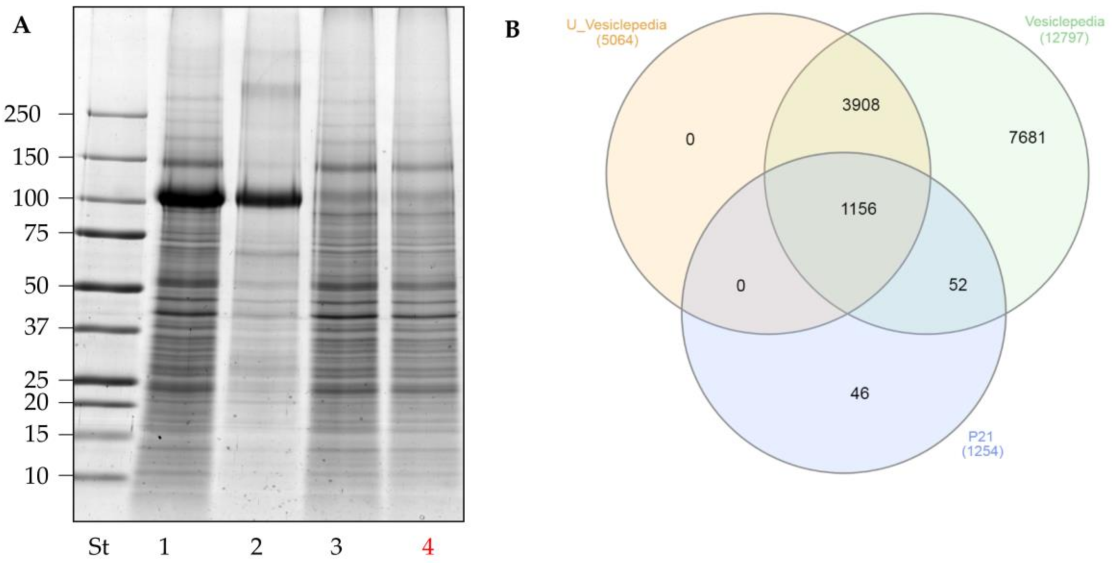

Supplementary Figure S8 Quality check of the sample before MS analysis and comparative analysis with vesiclepedia data sets. SDS-PAGE electrophoresis was carried out to check protein pattern of P21 (lane 1), SN21 ^{TCEP} (lane 2); P21^{TCEP} (lane 3) and P21^{TCEP} after chloroform methanol delipidation (lane 4). Delipidated samples was forward to the proteomic facility at the University of Virginia for analysis (Panel A). Venn diagram generated by InteractiVenn tool (www.interactivenn.net) to compare our identifications (P21^{TCEP}) with the list of EVs proteins in vesiclepedia repository (Vesiclepediaa) (Version 4.1, 15 August 2018; http://microvesicles.org/) and a subset of urinary EVs proteins (U_Vesiclepedia).
(http://microvesicles.org/browse_results?org_name=&cont_type=&tissue=Urine&gene_symbol=&ves_type=).

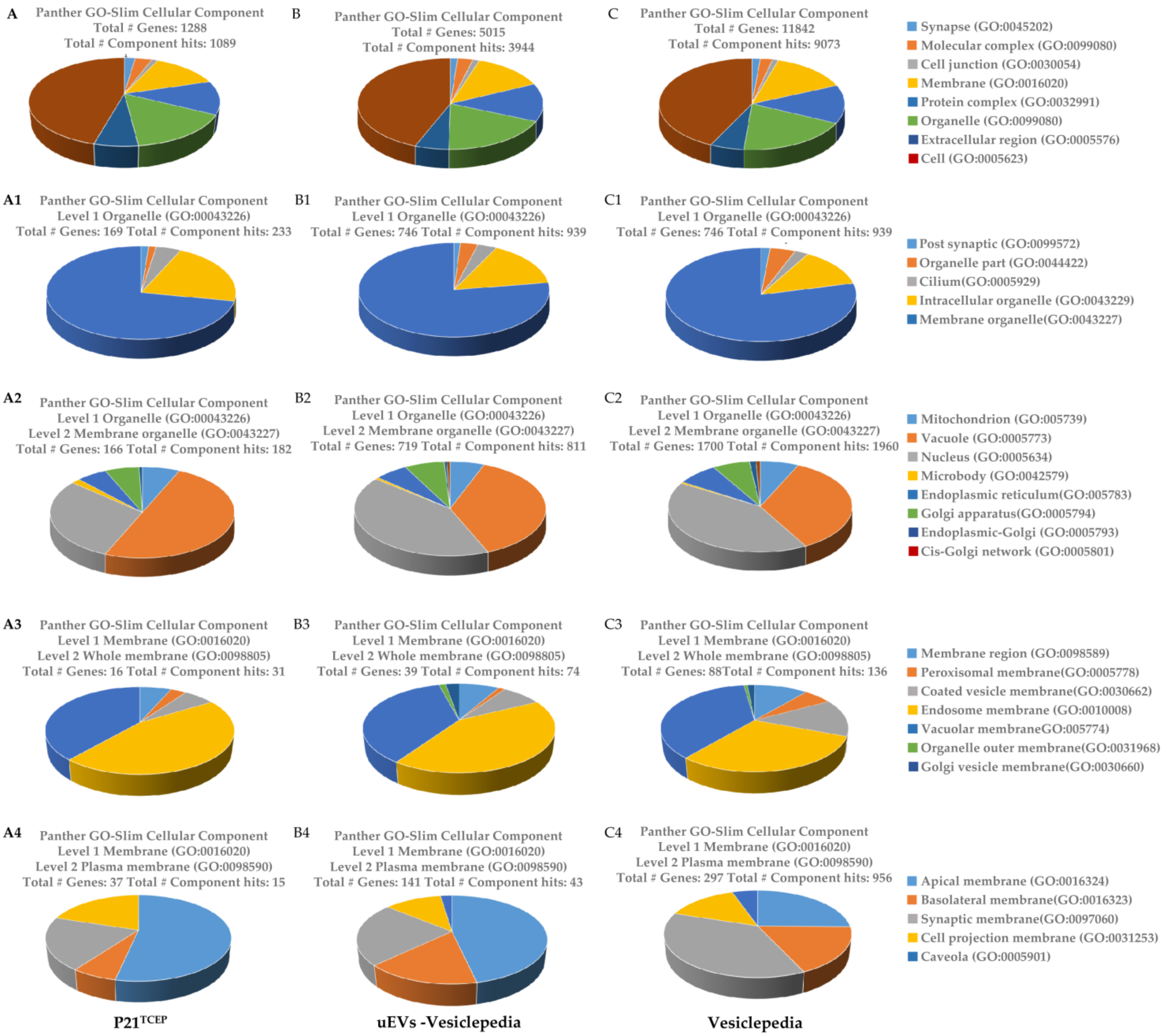

Supplementary Figure S9. Go annotation of EVs gene name protein by Panther classification for cellular component. Column A-A4 uEVs identifications in $P21^{TCEP}$; Column B-B4 identifications from uEVs extrapolated from vesiclepedia data set (uEVs-vesiclepedia). Column C-C4 identifications from EVs extrapolated from vesiclepedia data set (vesiclepedia).

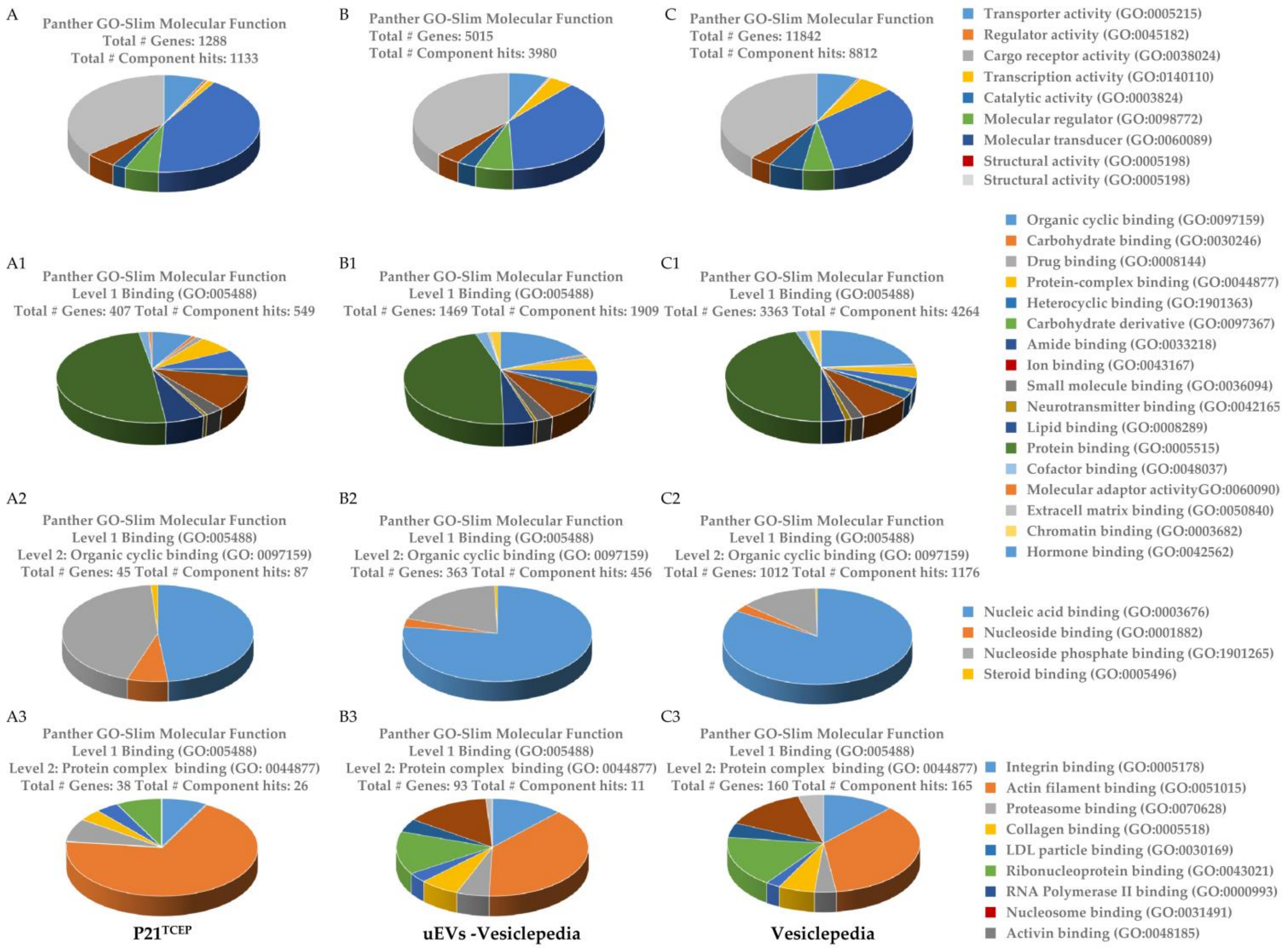

Supplementary Figure S10 Go annotation of EVs gene name protein by Panther classification for molecular sub fractions. Column A-A4 uEVs identifications in P21^{TCEP}; Column B-B4 identifications from uEVs extrapolated from vesiclepedia data set (uEVs-vesiclepedia). Column C-C4 identifications from EVs extrapolated from vesiclepedia data set (vesiclepedia)

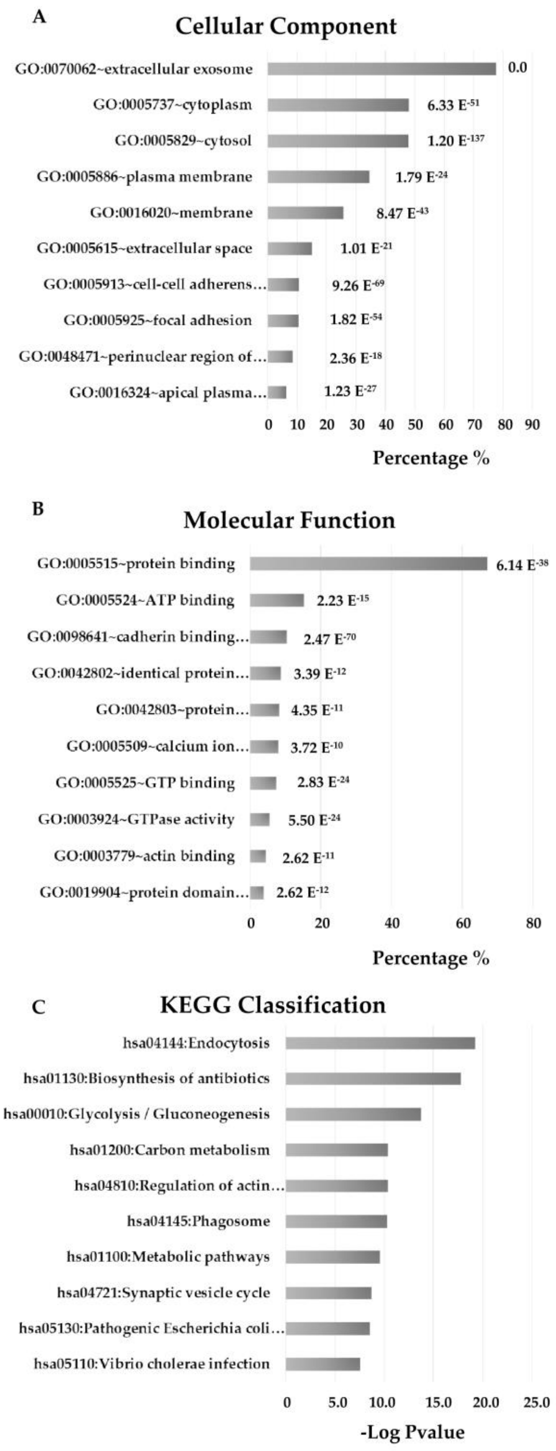

Supplementary Figure S11 GO annotation of uEVs $P21^{TCEP}$ data set by DAVID algorithm. Proteins identified in the $P21^{TCEP}$ dataset were used for enrichment analyses. Panel A Cellular component Panel B Molecular function and Panel C KEEG pathway enrichment. The top 10 categories are represented. The full list is available in Supplementary table S6

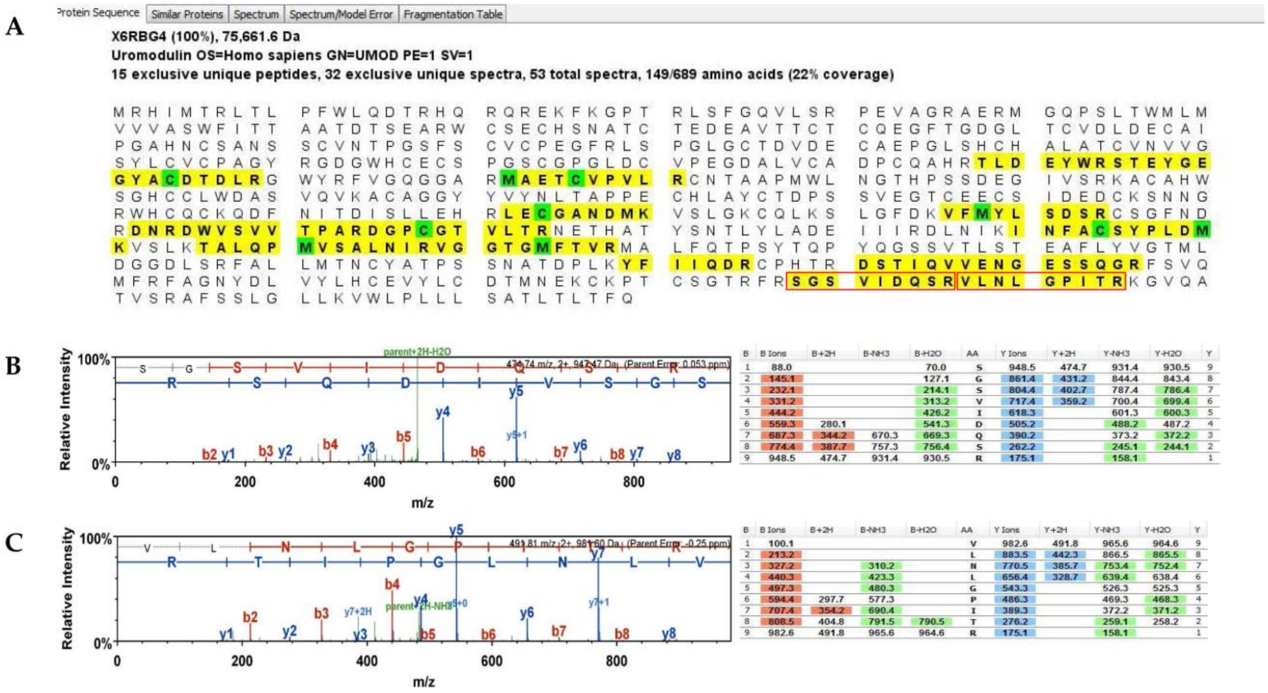

Supplementary Figure S12 Tamm Horsfall Protein identified unique peptides and MS/MS **spectra.** Panel A; Extrapolation the THP sequence from scaffold. Identified unique peptides are highlighted in yellow. Panel B and C MS/MS spectrum of the peptides between proteolytic cleavage of serine S in position 589 and the serine in position 614 bound to the glycophosphatidylinositol (GPI) anchor.

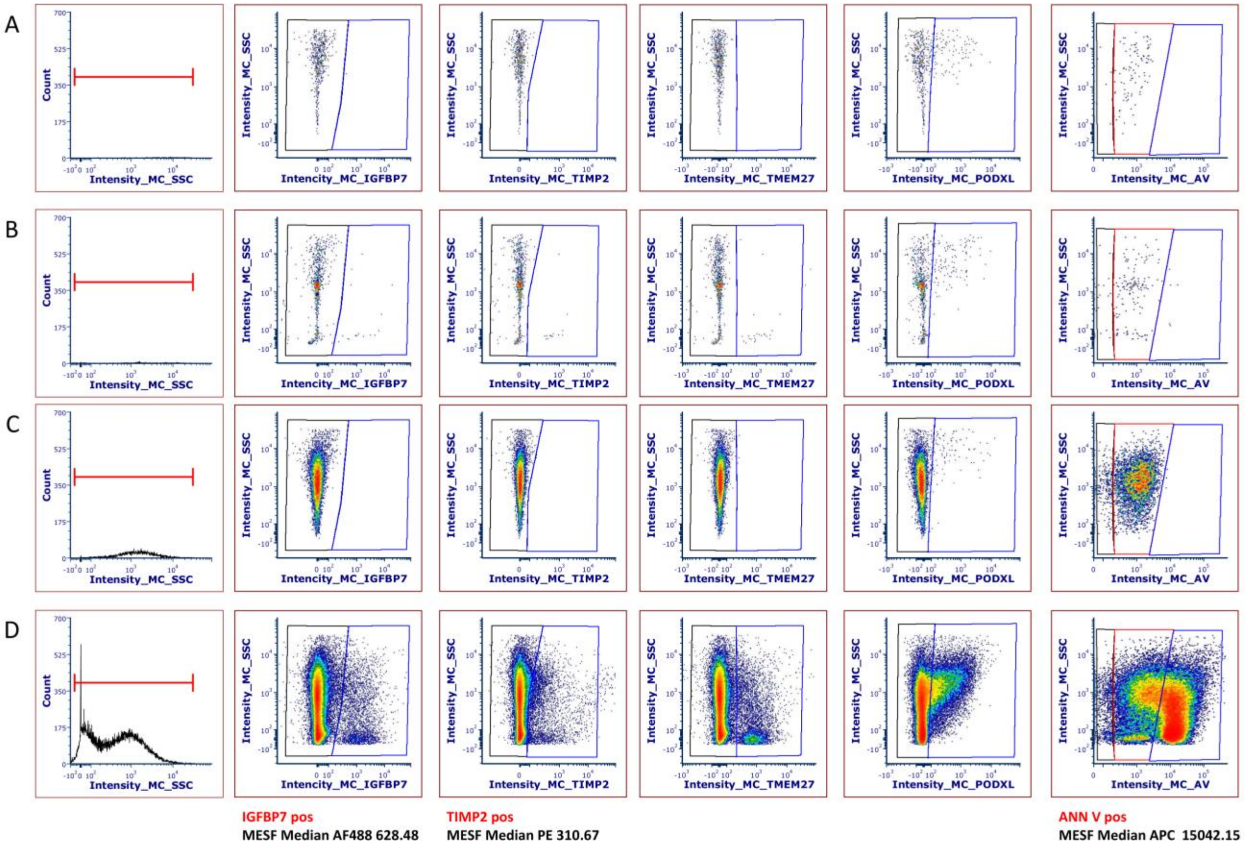

Supplementary Figure S13 Gate strategy and negative controls. Dot plots show all the event detected in Buffer only (row A), buffer plus reagents (row B), uEVs only (row C) and uEVs plus reagents (row D).

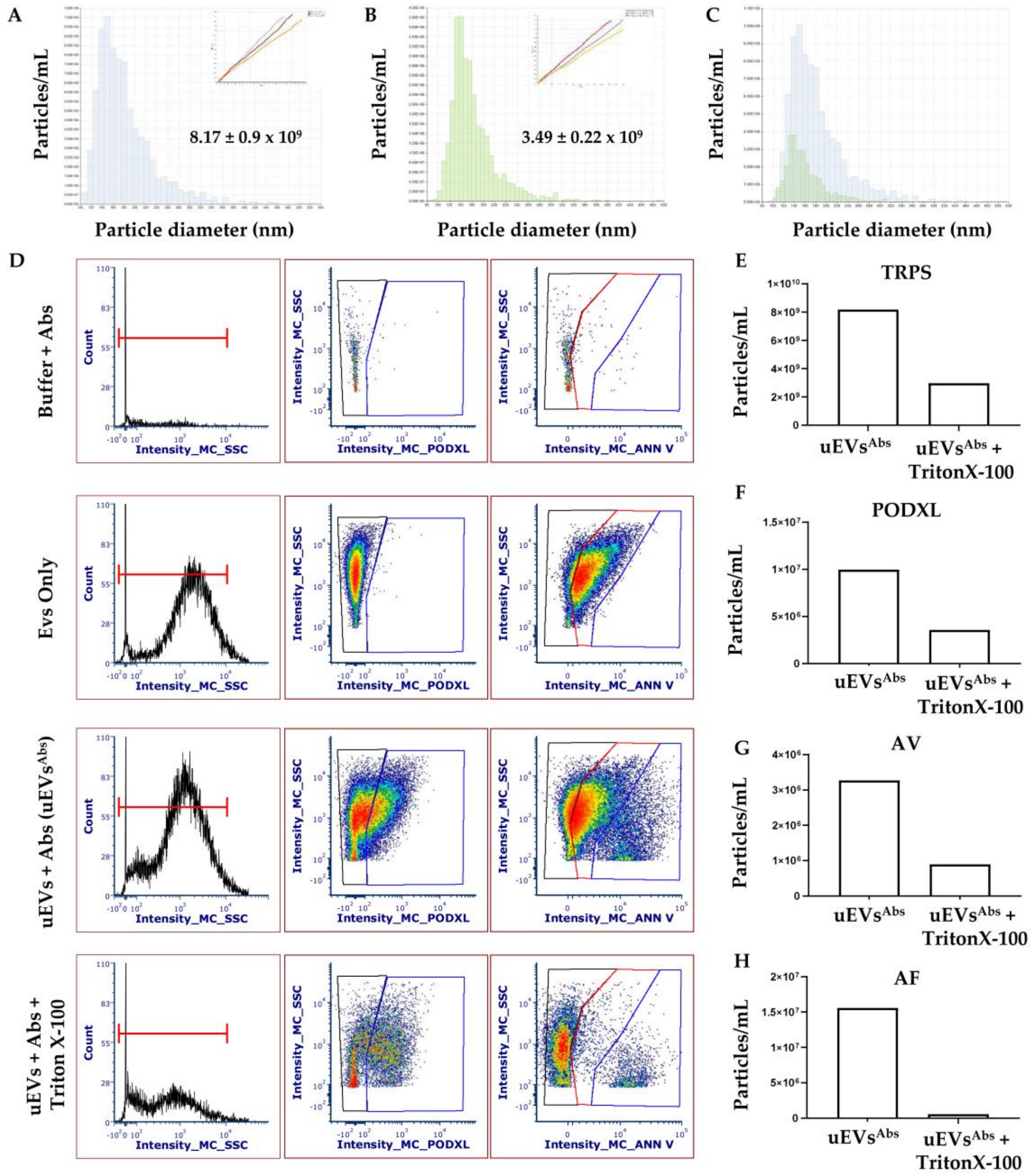

Supplementary Figure S14 Detergent lysis. Tunable resistive pulse sensing analysis of uEVs before detergent lysis (A) and after incubation with 0.8 % Triton X100 (B). Insert in the upper right corner shows the particle rates recorded for 3 different pressure. Overlap of PSD measured in TRPS (C) and particle concentration measured in TRPS (E) and flow cytometry (D, F,G and H) before and after detergent lysis for Podocalyxin (PODXL: F); Annexin V (AV: G) and auto fluorescence (AF: H).

A

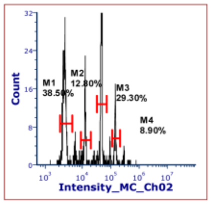

AF488				
	Median	MedFI	log Median	log MedFI
Pick 1	2856.42	3179	3.4558	3.5023
Pick 2	12614.10	22718	4.1009	4.3564
Pick 3	45733.55	95426	4.6602	4.9797
Pick 4	140667.90	333766	5.1482	5.5234

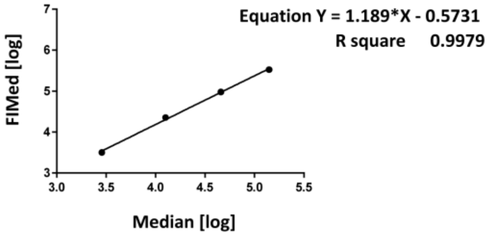

B

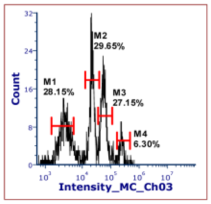

PE				
	Median	MedFI	log Median	log MedFI
Pick 1	2854.66	2481	3.4556	3.3946
Pick 2	21945.00	22212	4.3413	4.3466
Pick 3	55386.34	58449	4.7434	4.7668
Pick 4	235069.95	266267	5.3712	5.4253

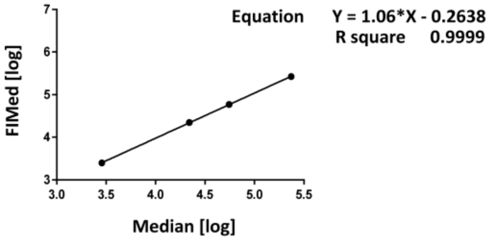

C

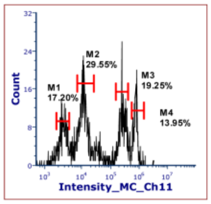

APC				
	Median	MedFI	log Median	log MedFI
Pick 1	2731.94	20993	3.4365	4.3221
Pick 2	11932.86	137581	4.0767	5.1386
Pick 3	263486.38	725533	5.4208	5.8607
Pick 4	769202.00	2671841	5.8860	6.4268

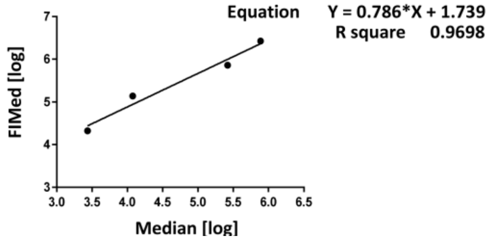

Supplementary Figure S15 MESF calibration beads. The median fluorescent intensity of each peak of the MESF for Alexa Fluor 488 (A) phycoerythrin -(B) and allophycocyanin (C) were used to calculate a linear regression analysis for estimating MESF values of uEV samples for IGFBP7, TIMP2 and AV respectively. The slope (x) and the (y) intercept were used to calculate the MESF value of the unknown samples.

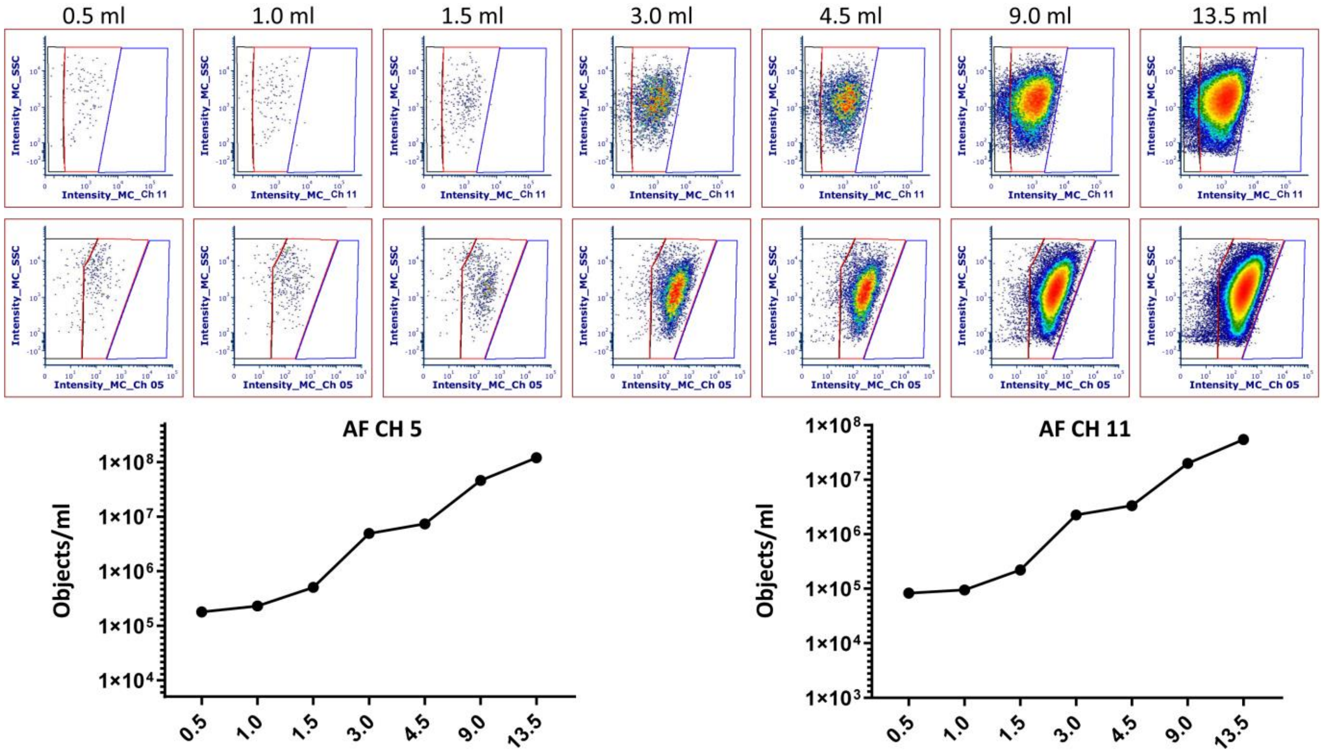

Supplementary Figure S16 Auto-fluorescence of uEVs without reagents. Autofluorescence emission increased proportionally to the volume used to enrich uEVs for both channel 5 and 11

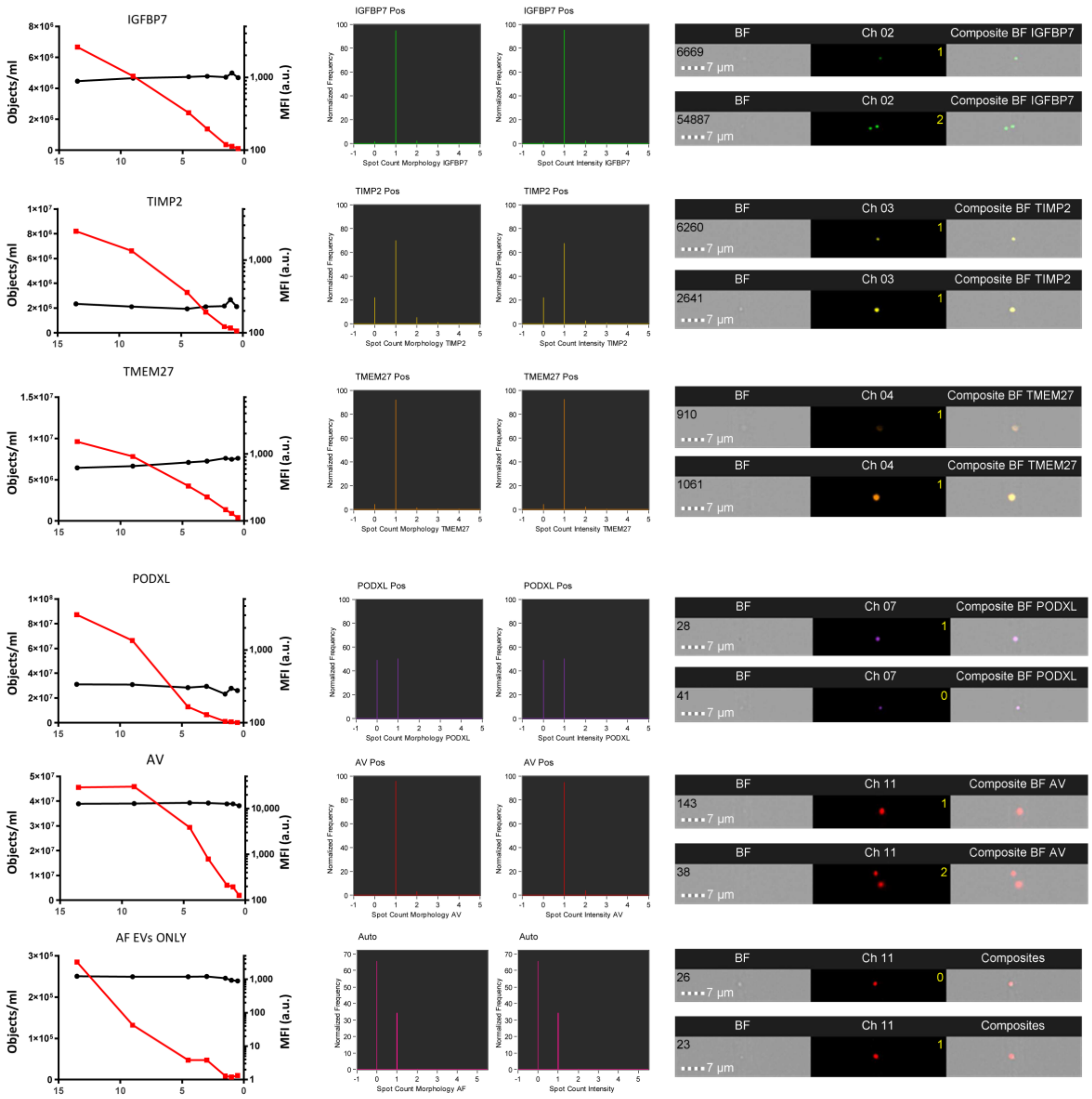

Supplementary Figure S17 Dilution analysis to evaluate "swarming" effect. The median fluorescence intensity and the particle concentration for each fluorecence dye and antigen and AF was plotted with respect to the amount of uEVs obtained from different volume of urine (x axe volume of urine processed). For the highest value the Boolean or logic masking algorithm was creating both a morphology and intensity mask combined with the spot count feature was created to further assess potential swarming. Some image gallery representative are show with the yellow number indicating the number of object identified.

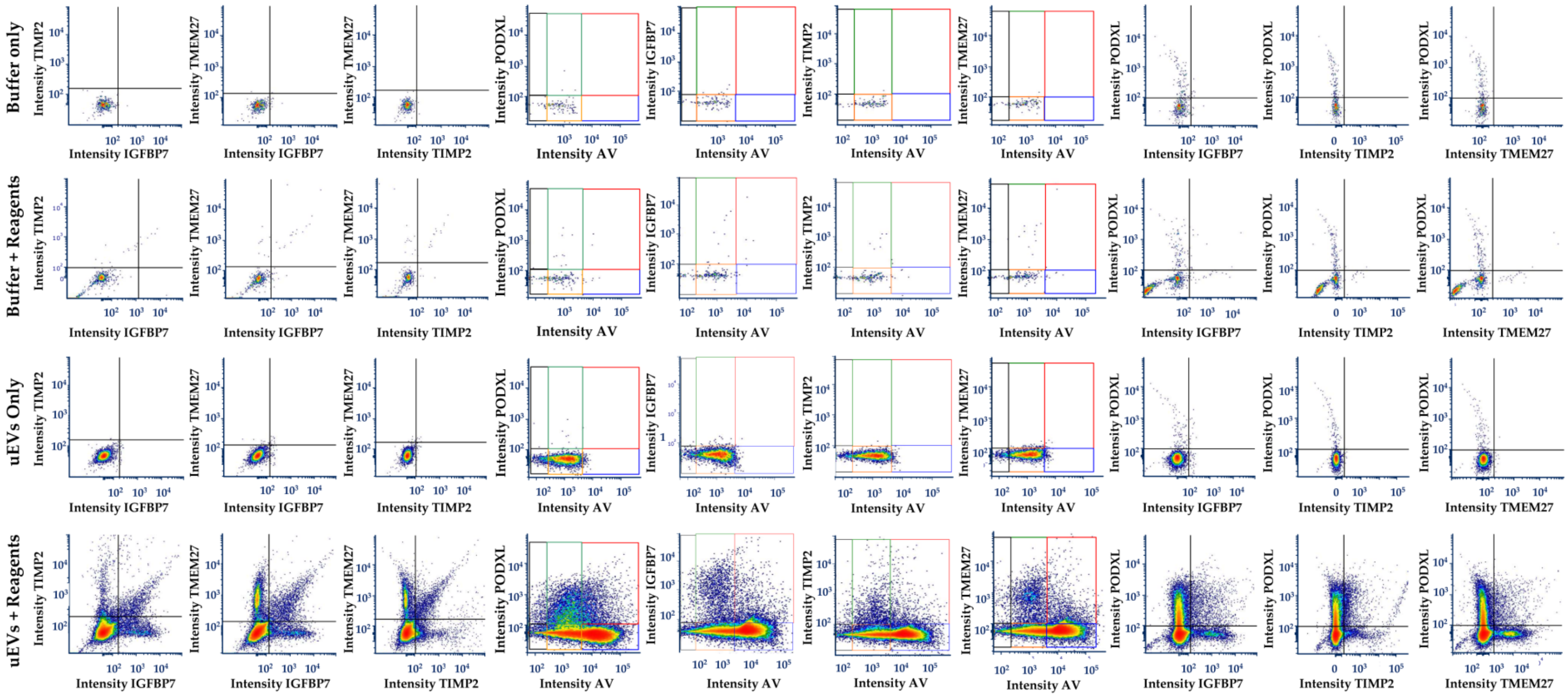

Supplementary Figure S18 Analysis of uEVs double staining. All the potential double combination were created to identified particles carrying more than one antigen.

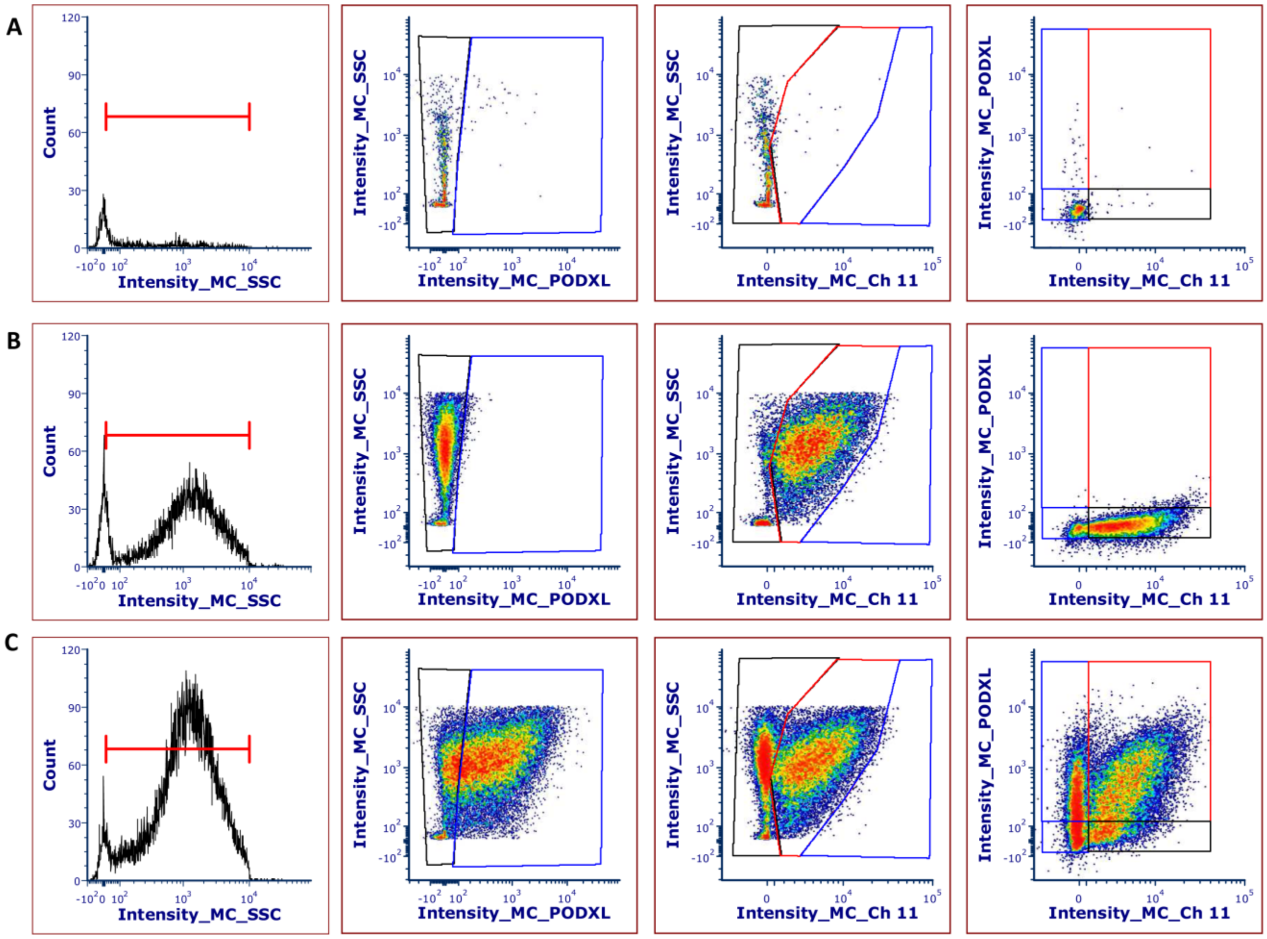

Supplementary Figure S19 PODXL single staining and co-detection with auto-fluorescence.

A Buffer plus anti PODXL antibody. B uEVS only C uEVs plus anti PODXL antibody

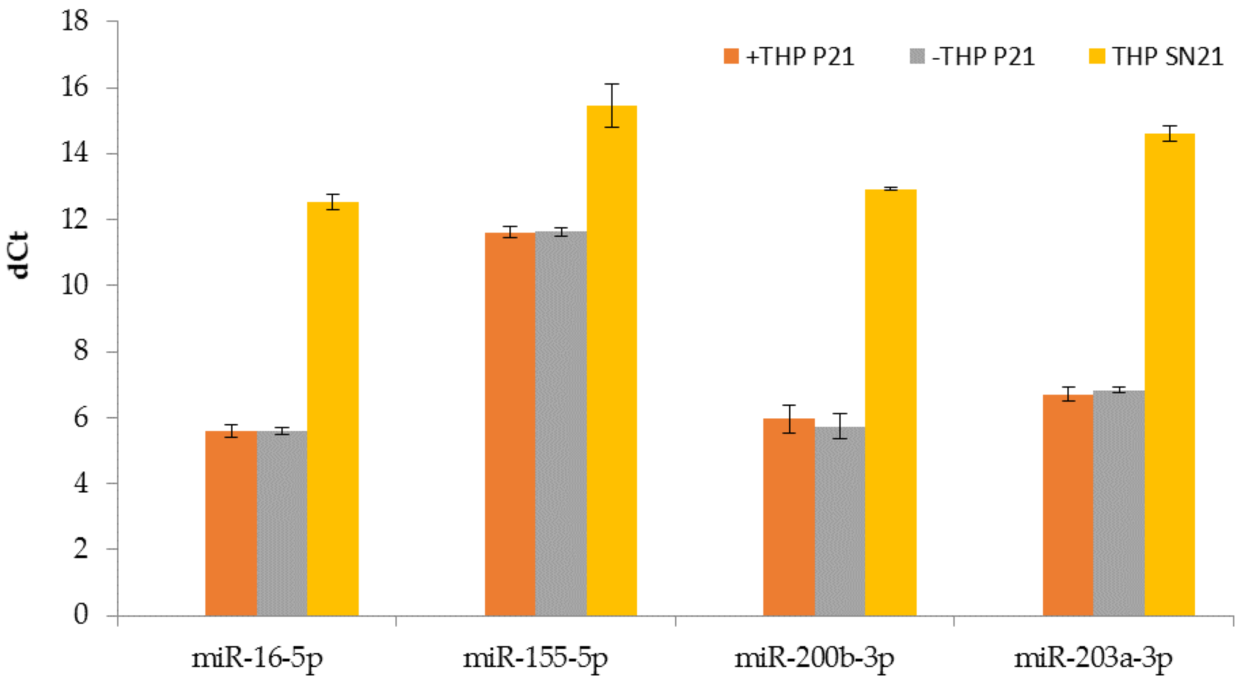

Supplementary Figure S20: No quantitative difference in miRNA content observed after treatment with TCEP to remove THP. The miRNA quantity in P21 from the same urine sample was tested with (-THP P21^{TCEP}) and without (+THP P21) TCEP treatment. Total RNA was isolated and Quantitative real time PCR was done for miR-16-5p, miR-155-5p, miR200b-3p, miR-203a-3p and cel-miR-39 (Spike-in). The cycle threshold values were normalized against the spike and were plotted in the y axis. The same was also done for the SN21^{TCEP} fraction after TCEP treatment to compare the miRNA content with P21 and P21^{TCEP}. No significant differences in measured miRNA content were observed between the –THP P21^{TCEP} and +THP P21 fractions.
